# Assessing the success of hydrological restoration in two conservation easements within Central Florida ranchland

**DOI:** 10.1371/journal.pone.0199333

**Published:** 2018-07-03

**Authors:** Grégory Sonnier, Patrick J. Bohlen, Hilary M. Swain, Steve L. Orzell, Edwin L. Bridges, Elizabeth H. Boughton

**Affiliations:** 1 Archbold Biological Station, Venus, Florida, United States of America; 2 University of Central Florida, Orlando, Florida, United States of America; 3 Avon Park Air Force Range, Avon Park, Florida, United States of America; 4 Botanical and Ecological Consultant, Bremerton, United States of America; Chinese Academy of Forestry, CHINA

## Abstract

In the USA, the United States Department of Agriculture (USDA) Natural Resource Conservation Service (NRCS) has restored millions of acres of wetlands through its Wetland Reserve Easement (WRE) programs. However, few quantitative studies have explored whether WREs have enhanced wetland hydrology and wetland plant communities. Additionally, USDA Compatible Use Permits for cattle grazing and other management practices are sometimes issued for WREs, but little is known about potential benefits/detriments of such practice on the success of wetland restoration. In this study, we tested if hydrological restoration of previously drained species poor pastures increased water depth and hydroperiod. Restoration involved plugging key ditches, adding water control structures and a berm. We also tested if hydrological restoration increased plant diversity (alpha and beta), floristic quality (using coefficient of conservatism) and increased the cover of wetland species (using species wetland status). Finally, we tested if cattle grazing had an effect on the success of restoration by comparing grazed plots to fenced plots. We studied two conservation easements (a total of 748 acres) located on semi-native pastures in central Florida, USA. We monitored vegetation using permanent transects stratified by vegetation type before (2004–2005) and after (2012) the restoration (2008). We assessed wetland hydroperiod using groundwater wells set up in 2003 and located within and outside the boundaries of these two easements. We used linear mixed models and multivariate analyses to compare vegetation and hydroperiods pre- and post-restoration. Number of flooded days increased following restoration in one of the easements, but we did not detect significant changes in hydrology in the other easement. Floristic quality, beta diversity and cover of obligate wetland species increased in both conservation easements and in most vegetation types. These vegetation changes were likely due to restoration activities since annual rainfall was not significantly different pre- and post-restoration. Cattle grazing did not have a negative or positive effect on the success of restoration, nor did we detect any positive effect of grazing on the success of restoration. Overall, our study shows that hydrological restoration can enhance wetland hydroperiod, water depth and wetland vegetation, but more resources should be allocated to short- and long-term monitoring of the restoration success.

## Introduction

Wetlands occupy only 6–9% of the landscape worldwide, but they have a large influence on ecological functions in the landscape [1–3] and provide multiple ecosystem services whose economic value is immense [4–6]. For example, they provide hydrologic services by acting as “sponges” storing water and slowly releasing it, thus reducing flood heights and costly damage following storms [7]. Wetlands are efficient water filtration systems improving water quality. Wetlands also support a high biodiversity as they are a crucial habitat for flora and fauna (nesting/reproductive habitat, and forage habitat) [8,9]. Despite their importance, the loss and degradation of wetlands has been considerable [2,3,10,11] and more rapid than for other ecosystems [12]. One reason is that wetlands are often perceived negatively because of poor understanding of wetland environmental and economic values. Losing wetlands implies losing crucial biological functions, especially when these wetlands are isolated and connectivity among wetlands is reduced [13].

In response to extensive wetland losses, policy makers have made efforts to i) preserve existing wetlands, ii) mitigate the loss of wetlands and iii) restore degraded or lost wetlands. The Wetland Reserve Easement (WRE) program (formerly known under the Wetland Reserve Program, WRP), created in 1985 by the Natural Resources Conservation Service (NRCS), United States Department of Agriculture (USDA) is one such effort. The Wetland Reserve Easement (WRE) program is a voluntary program that provides technical and financial support to public and private landowners in their efforts to protect, restore and enhance wetlands. When enrolled in the program landowners retain ownership of their land, but they agree to limit its future use, such as cattle grazing. The overarching goal of the WRE program is to restore the hydrology of a site as close as possible to pre-human development condition, in order to improve wetland habitat for wildlife. However, this goal is often tempered by economic feasibility and the ways to achieve this goal are dependent on the history and geography of the site being restored.

The USDA Conservation Effects Assessment Project (CEAP) was initiated in 2003 to scientifically quantify the environmental effects of conservation practices [14,15]. DeSteven and Lowrance (2011) found little study of the effectiveness of conservation practices within agricultural lands in the U.S. Coastal Plain and Piedmont region. In wetlands on agricultural land, hydrology is restored by drainage cessation and ditch plugging [16]. However, in Florida, flat areas and sandy soils create challenges to common restoration practices. How restored wetlands are managed is another key to success. The issue of cattle grazing in conservation easements is particularly controversial [17]. Cattle grazing has been found to degrade water quality in wetlands and alter plant community structure [18–22] with potential negative effects on wildlife [23,24]. In contrast, some studies showed potential benefits of grazing disturbance at low stocking densities, including reduction of invasive plants [25], maintaining diversity [26,27] and maintaining longer hydroperiod [26,28,29]. However, the effect of cattle on wetland restoration following NRCS guidelines has not been quantitatively assessed.

Here, we study the success of wetland restoration in two conservation easement sites located on a cattle ranch in central Florida. The first site (hereafter “East Marsh”) was historically a poorly drained wet palm savanna with a deeper sawgrass marsh in its center. Historical aerial photo (<1950) of the second site (hereafter “South Marsh”) showed that the landscape was composed of both a swamp (dominated by sweet bay *Magnolia virginiana*) and shallow marshes. Both sites were converted to pastures between 1950 and 1980. Sites were drained using a network of ditches, gradually cleared and planted with highly productive grass species (the most common being bahiagrass, *Paspalum notatum*). The goal of the restoration in these two easements was to increase water depth and hydroperiod to a level at least similar to typical wet prairie in Florida (>50 days of flooding per year), without affecting pastures adjacent to the easements. This was done by plugging key ditches, adding water control structures and adding a berm in one of the easements. No tree or native species planting was implemented in the restoration process, because of the cost of planting at such large scale. Thus the restoration goal laid out by NRCS for plant communities was not to revert to exact historical communities (swamp, wet savanna), but instead to increase diversity, floristic value and the cover of wetland species such as sawgrass (*Cladium jamaicense*) and maidencane (*Panicum hemitomon*) while decreasing the cover of bahiagrass (*Paspalum notatum*). Our objectives were threefold. First, we determined whether the hydrological restoration increased water table and hydroperiod. Second, we asked whether the restoration increased alpha and beta plant diversity, floristic quality, and the cover of wetland species such as sawgrass and maidencane, while decreasing the cover of upland species such bahiagrass. Maidencane and sawgrass were chosen as indicator species because they are two of the most common obligate wetland species in Florida. Bahiagrass was chosen as an indicator species, because it is the most common forage grass species in Florida, it is non-native to Florida and it often covers >75% of a plot in unrestored wetlands. Third, we assessed the effect of cattle grazing on the success of the restoration. Due to strict USDA guidelines allowing only low density grazing and potential benefits of intermediate disturbance, we expected no negative impact of cattle grazing on restoration success.

## Material and methods

### Study sites, historical communities and restoration design

**Fig 1:**
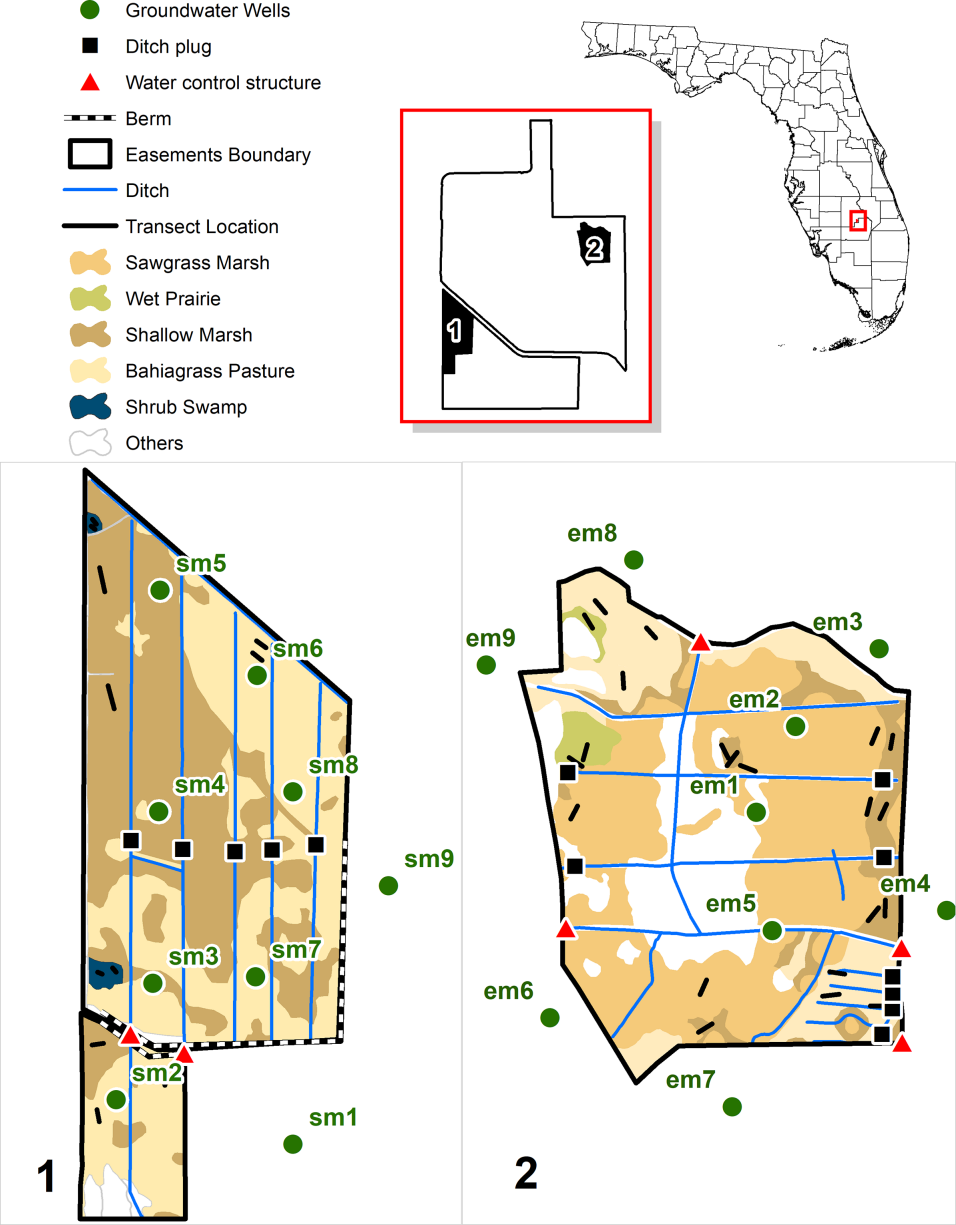
Location of the two conservation easements at Buck Island Ranch. Vegetation types and locations of the groundwater wells and vegetation transects are shown in the South Marsh (1) and the East Marsh (2).

Study sites consist of two USDA WREs located at Buck Island Ranch in south central Florida. Buck Island Ranch is a full-scale working cattle ranch functioning as a cow-calf operation, as well as a biological field station (MacArthur Agro-ecology Research Center, a division of Archbold Biological Station). Historically (<1950), the landscape of the South Marsh (470 acres, Fig 1) was composed of a closed canopy forested seepage wetland (dominated by sweet bay, *Magnolia virginiana*) and open shallow marshes that were rarely penetrated by wildfires. The East Marsh (280 acres, Fig 1) was formerly a mosaic of pyrogenic community types including wet prairie, sawgrass marsh, and palm and cordgrass savanna typical of the “Indian Prairie” region of Florida [30].

Both easements were converted to pastures between 1950 and 1980. Wetlands were drained through a network of ditches; the South Marsh was cleared; and non-native highly productive grass species were planted to improve forage quality (*e*.*g*. Bahiagrass *Paspalum notatum*). The South Marsh easement was more highly modified with more intense drainage and more complete planting compared to the East Marsh easement. After conversion, the South Marsh was dominated by a mixture of native and non-native grasses with isolated patches of herbaceous wetland plants [30]. Bahiagrass cover was particularly important (>50%) throughout the South Marsh. Following conversion, the East Marsh easement contained most of its native components except for conversion of some patches of the driest areas into non-native bahiagrass pasture. Both easements are embedded within semi-native pastures, which are typically lightly drained, have not been fertilized, and vegetation composition still contain a large proportion of native species [31]. This contrasts with highly modified improved pastures on the ranch that have been fertilized, extensively drained, with native vegetation completely replaced by non-native and highly productive forage grasses [31].

The restoration design and plans were established in the early phase of the project by a subcontracted engineering company, and after discussion with NRCS representatives. The goal of the restoration was to increase water levels and hydroperiod but not to create large ponded wetlands. As such, the design sought to retain rainfall and prevent surface flow from leaving the easement via major ditches during the wet season, while allowing the site to dry down during the dry season. To achieve this, we plugged key ditches within each easement and added a series of water control structures at major outflow locations (Fig 1). Ditches were not backfilled because of cost and risk of introducing non-native species. Finally, a berm was built to prevent flow from the pasture to the largest ditch in the South Marsh. One major constraint of the design was that wetland restoration should not affect water level outside of the easement. Restoration constructions occurred in 2008 during the dry season. The restoration plans initially included native tree planting in the South Marsh, but it was not implemented due to costs.

### Monitoring rainfall and hydrology: Design and metrics

We obtained daily rainfall data from a weather station located on the ranch, three miles away from each easement. We extracted data from 2000 to 2014 and calculated annual rainfall for each year. To follow changes in ground/surface water level through time, groundwater wells (GDWs) were installed in each of the two conservation easements in 2003 (Fig 1). In this study, we used well data for the period between 2003 and 2014. The restoration occurred during 2008, providing five years of pre-restoration and six years of post-restoration data. We established six GDWs within the South Marsh easement and three outside the South Marsh for a total of 9 wells (Fig 1). In the East Marsh easement, we established 3 GDWs within the boundaries of the easement and 6 outside the easement, again for a total of 9 groundwater wells (Fig 1). Groundwater levels were recorded using non-vented pressure transducers placed within each well (Level TROLL^®^ 300, rugged TROLL^®^ 100 Data Loggers, In-Situ Inc.). We downloaded the data from these water level data loggers every 6–12 months in order to follow groundwater level through time. Groundwater level was corrected for barometric pressure using pressure data from a pressure transducer (Level TROLL^®^ 300, rugged TROLL^®^ 100 Data Loggers, In-Situ Inc.) suspended in one well above water and using the baro-merge process in Win-Situ Software. Groundwater well elevations were obtained through topographic surveys by a subcontracted engineering company. Water levels were expressed relative to elevation above mean sea level. Based on these groundwater well data, we estimated median water level depth (cm relative to well elevation) and the total number of flooded days per year. We calculated these variables for each well. Our dataset included missing data due to transducer malfunctions. For this reason, we removed annual median water level and annual hydroperiod estimates that had more than 30 days of missing data from our analyses.

### Plant community surveys: Design and metrics

We surveyed plant communities in each of the two conservation easements using permanently located transects and quadrats. These transects/quadrats were located within 5 major vegetation types (Fig 1, Table 1). Vegetation was surveyed between December 2004 and January 2005 (3 years before restoration) and later resurveyed in 2012 between February 7^th^ and February 20^th^ (4 years after restoration). We recorded the presence and cover (to the nearest 5%) of all species found in each 1m^2^ quadrat. Overall our dataset included 300 plots. Following the first survey, we established 19 10*10m exclosures along selected transects, in both the South Marsh and the East Marsh easements. Each exclosure included two sampled plots for a total of 38 ungrazed plots (28 in East Marsh and 10 in South Marsh). No control transects/quadrats were setup outside of the easements due to logistic constraints and lack of appropriate control sites with similar vegetation and hydrology.

**Table 1:** Details on the vegetation sampling in each of the conservation easement.

Easements	Vegetation type	Number of transects (quadrats)
South Marsh	Bahiagrass pasture	4 (40)
South Marsh	Shallow marsh	3 (30)
South Marsh	Shrub swamp	4 (20)
East Marsh	Bahiagrass pasture	6 (60)
East Marsh	Shallow marsh	6 (60)
East Marsh	Sawgrass marsh	6 (60)
East Marsh	Calcareous wet prairie ecotone	3 (30)

We analyzed five different plant community assemblages (Fig 1), namely bahiagrass pasture, shallow marsh, shrub swamp, sawgrass marsh and wet prairie on calcareous soil (hereafter, wet prairie). Bahiagrass pastures were highly modified pastures with at least 50% ground cover dominance of bahiagrass (*Paspalum notatum*), carpet grasses (*Axonopus* spp.), torpedo grass (*Panicum repens*), or other planted pasture grasses. Shallow marsh communities were diverse communities with over 50% native grasses (*e*.*g*. *Panicum hemitomon*), sedges (*e*.*g*. *Rhynchospora inundata*), and forbs, and less than 10% sawgrass (*Cladium jamaicense*) cover. Sawgrass marsh communities were dominated by *Cladium jamaicense* with at least 50% of total cover. Shrub swamp communities were remnant of a bay swamp community with greater than 50% cover of trees and shrubs, including *Magnolia virginiana*. Wet prairie communities were the most diverse plant communities including co-dominant species such *Muhlenbergia sericea*, *Schizachyrium rhizomatum*, *Eriochloa michauxii*, *Fimbristylis spadicea*.

We compiled native/non-native status from the literature (https://plants.usda.gov, http://florida.plantatlas.usf.edu). Based on this information and survey data, we calculated species diversity, exponential of Shannon diversity (H’) and cumulative cover of non-native species at the plot level. We also compiled the wetland indicator status of each species in our dataset from the literature (https://plants.usda.gov, http://florida.plantatlas.usf.edu). Wetland indicator status separates species in five broad categories. Obligate Upland species (UPL) and Facultative Upland species (FACU) prefer upland habitats and almost never or occasionally occur in wetlands. Facultative species (FAC) have no preference and occurred in both upland and wetland habitats. Obligate Wetland species (OBL) and Facultative Wetland species (FACW) almost always or usually occur in wetlands. Based on this information and survey data, we calculated the cumulative cover of each wetland status category at the plot level. Finally, we used coefficient of conservatism to calculate a floristic quality index. Coefficient of conservatism is a measure of plant fidelity to specific habitats and plant tolerance to disturbance, and it separates ubiquitous species (low coefficient of conservatism) from habitat specialists (high coefficient of conservatism). We used the classification proposed by Montellaro *et al*. [32] organized on a 1–10 scale and attributed a 0 value to non-native invasive species. We estimated floristic quality as the (weighted) mean coefficient of conservatism observed in each plot.

### Statistical analysis

Using daily rainfall data, we compared annual rainfall patterns between 2000 and 2005 (*i*.*e*. before the first survey) and between 2006 and 2011 (*i*.*e*. between the two surveys) using t-test. We analyzed the South Marsh and East Marsh data separately. We did not expect a similar response to hydrological restoration, because these two easements were within two separate watersheds, restored with different designs and had different land use legacies before restoration. All analyses were performed in R software. To address our first objective, we related the number of flooded days per year to hydrological restoration using linear mixed models with well ID as random factor to account for non-independence of the data coming from the same well. Wetland hydroperiods at Buck Island Ranch are primarily driven by rainfall and wetlands at higher elevation can drain more quickly and have shorter hydroperiods [33]. For these reasons, we used annual rainfall and groundwater well elevation as covariates in our linear mixed models We repeated this analysis with median water depth (relative to ground surface) as a response variable. These analyses were performed using the *nlme* package [34].

To determine if restoration had a significant effect on vegetation, we further divided our dataset and analyzed each vegetation type separately. We expected the vegetation response to restoration to vary between community types. We related each diversity metric to restoration using a generalized linear mixed model since our design included both nested (plots within transects) and repeated data (permanent plots and transects). Response variables were species richness, H’ (exponential of Shannon diversity), non-native species cover, maidencane, bahiagrass, and sawgrass cover, floristic quality and cover of obligate wetland species as well as cover of UPL, FACU, FAC and FACW species. We used non-metric multi-dimensional scaling (NMDS) with Bray-Curtis distance to observe changes in community composition. We tested if composition shifted following restoration using permutational multivariate analysis of variance [35]. Finally, we tested for differences in beta-diversity before and after restoration using the homogeneity of multivariate dispersion test [36,37]. This method is based on ecological distances between plots (in our case Bray-Curtis distance) and quantifies beta diversity as the average distance between group members (*e*.*g*. plot 1 in bahiagrass pasture community before restoration) to the group centroid (*e*.*g*. centroid of all plots in bahiagrass pasture community before restoration). These tests were performed using the “adonis” and “betadisper” functions respectively, available in the Vegan Package [38].

To determine how grazing affected restored communities, we compared fenced plots (n_fenced_ = 38) to the closest grazed plots from the same transect (n_grazed_ = 38). We related species richness, non-native cover, grass cover, tree and shrub cover to grazing using linear mixed models with plots within transects as random effect and only post-restoration data.

## Results

We compared annual rainfall between 2000 and 2005 (*i*.*e*. before the first survey) and between 2006 and 2011 (*i*.*e*. between the two surveys). We found that annual rainfall was not significantly different with on average 109.3±27.7cm before the first survey and 111.2±22.5cm between the two surveys (t = -0.13, df = 9.59, p = 0.9).

### Restoring hydrology

The number of flooded days per year and median water table increased following restoration in the South Marsh easement (Table 2, S1 Fig). For a similar amount of rainfall, the South Marsh experienced, on average, 20.16 CI[0,44.60]flooded days per year pre-restoration and 95.11 CI[73.24,116.97] flooded days per year post-restoration (representing close to 5-fold increase). This effect was stronger at intermediate elevations and 6 out of 7 wells within the South Marsh WRE showed an increase in number of flooded days/year (S1 Fig). The only exception was well SM2 located in a different “watershed sub-basin” and at a higher elevation. We observed a tendency for higher number of flooded days and higher median water table following restoration outside of the South Marsh easement. However, this increase was not significant and mainly due to well SM9.

**Table 2:** Results of linear mixed models testing the effect of restoration on hydrology, outside and inside the boundaries of each easement. Each model included well elevation and annual rainfall as covariates.

Easements	Response variables	Explanatory variables	Chisq	P-value
**South Marsh**	Flooded days/year	Restoration	26.38	**<0.001**
**(within easement)**		Annual rainfall	3.77	0.052
		Well elevation	8.39	**0.004**
	Pre-restoration	Post-restoration		
	20.2 CI [0, 44.6]	95.1 CI [73.4, 117.0]		
**South Marsh**	Median Water table	Restoration	68.38	**<0.001**
**(within easement)**	(annual)	Annual rainfall	23.88	**<0.001**
		Well elevation	5.83	**0.016**
	Pre-restoration	Post-restoration		
	-95.2 CI [-105.6, -84.9]	-38.4 CI [-47.3, -29.5]		
				
**South Marsh**	Flooded days/year	Restoration	2.25	0.156
**(outside easement)**		Annual rainfall	1.97	0.182
		Well elevation	28.66	**<0.001**
	Pre-restoration	Post-restoration		
	50.4 CI [0, 104.4]	75.8 CI [23.2, 128.3]		
**South Marsh**	Median Water table	Restoration	1.77	0.205
**(outside easement)**	(annual)	Annual rainfall	6.51	**0.020**
		Well elevation	6.47	**0.023**
	Pre-restoration	Post-restoration		
	-74.4 CI [-115.5, -33.4]	-57.4 CI [-97.4, -17.4]		
** **				
**East Marsh**	Flooded days/year	Restoration	2.84	0.106
**(within easement)**		Annual rainfall	12.78	**0.002**
		Well elevation	1.35	0.257
	Pre-restoration	Post-restoration		
	220.6 CI [74.8, 365]	180.8 CI [37.2, 324.4]		
**East Marsh**	Median Water table	Restoration	1.22	0.28
**(within easement)**	(annual)	Annual rainfall	13.88	**0.001**
		Well elevation	1.45	0.24
	Pre-restoration	Post-restoration		
	2.7 CI [-75.6, 80.9]	-6.8 CI [-83.9, -70.3]		
				
**East Marsh**	Flooded days/year	Restoration	7.66	**0.006**
**(outside easement)**		Annual rainfall	14.07	**<0.001**
		Well elevation	29.79	**<0.001**
	Pre-restoration	Post-restoration		
	48.9 CI [30.8, 67.0]	24.5 CI [9.1, 39.9]		
**East Marsh**	Median Water table	Restoration	6.84	**0.009**
**(outside easement)**	(annual)	Annual rainfall	23.70	**<0.001**
		Well elevation	36.36	**<0.001**
	Pre-restoration	Post-restoration		
	-89.8 CI [-106.2, -73.4]	-110.5 CI [-124.6, -96.5]		

We did not observe any significant increase in the number of flooded days and median water level inside the East Marsh easement. We detected a significant decrease in number of flooded days and water table in wells located adjacent to the East Marsh (Table 2, S1 Fig).

### Restoring plant communities

**Fig 2:**
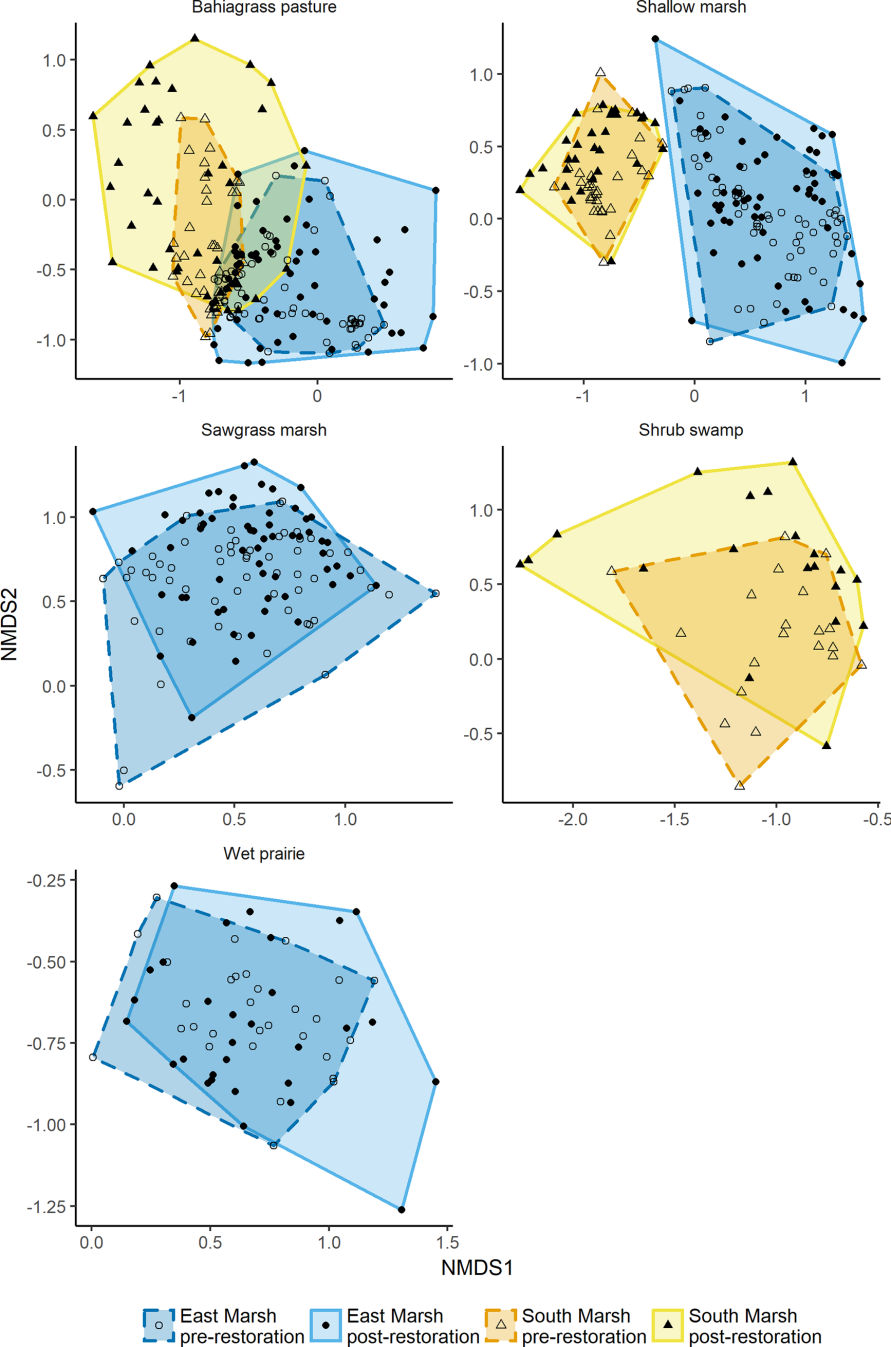
Non-metric multidimensional scaling showing shift in vegetation composition following restoration in each of the two conservation easements. The South Marsh easement contained bahiagrass pasture, shrub swamp, and shallow marsh, whereas the East Marsh easement contained bahiagrass, shallow marsh, sawgrass marsh, and wet prairie communities.

We observed that beta diversity increased in most communities after restoration, except the wet prairie community in the East Marsh (Table 3, Fig 2). Beta diversity significantly decreased in the sawgrass community of the East Marsh. Alpha (quadrat level) species richness varied among vegetation types and between the two conservation easements (Table 3). Species richness (measured at a 1 m^2^ scale) was on average lower in bahiagrass pastures (6.92±4.59 in the South Marsh and 7.08±3.04 in the East Marsh) and shrub swamps (6.80±2.36) and higher in wet prairies (17.17±4.32) before restoration. Species richness was higher in the East Marsh compared to the South Marsh (*i*.*e*. shallow marsh community in the East Marsh SR = 12.23±3.89 *vs*. the South Marsh = 7.80±3.01). Restoration had a significant negative effect on species richness in bahiagrass, and shrub swamp of the South Marsh and in shallow marsh and wet prairie of the East Marsh (Table 3, S2 Fig). No communities showed an increase in the number of species following restoration. When using the exponential of Shannon diversity (H’) we observed that diversity significantly decreased by 13.28% and by 20.20% in the sawgrass and shallow marsh communities respectively, but increased by 42.30% in the bahiagrass pasture community in the East Marsh.

**Table 3:** Results of linear mixed models testing the effect of restoration on each plant communities in the South Marsh (A) and in the East Marsh (B).

Community types	Metrics	pre restoration	post-restoration	F-value	P-value
**A) South Marsh**					
Bahiagrass pasture	SR	6.93	5.18	F1,39 = 7.78	**0.008**
	H'	2.60	2.94	F1,39 = 1.73	0.196
	Non-native cover	0.58	0.32	F1,39 = 14.14	**<0.001**
	Beta diversity	0.41	0.57	F1,79 = 32.69	**<0.001**
	Floristic Quality	2.42	2.81	F1,39 = 3.25	0.079
	OBL cover	0.04	0.27	F1,39 = 24.60	**<0.001**
	OBL+ FACW cover	0.10	0.35	F1,39 = 30.49	**<0.001**
	FACU+UPL cover	0.88	0.53	F1,39 = 53.53	**<0.001**
					
Shallow marsh	SR	7.80	6.17	F1,29 = 10.11	**0.004**
	H'	4.12	3.29	F1,29 = 5.50	**0.026**
	Non-native Cover	0.04	0.03	F1,29 = 1.99	0.169
	Beta diversity	0.43	0.54	F1,59 = 9.14	**0.006**
	Floristic Quality	3.23	4.28	F1,29 = 69.79	**<0.001**
	OBL cover	0.22	0.35	F1,29 = 11.58	**0.002**
	OBL+ FACW cover	0.47	0.69	F1,29 = 20.84	**<0.001**
	FACU+UPL cover	0.49	0.23	F1,29 = 33.75	**<0.001**
					
Shrub swamp	SR	6.80	4.60	F1,19 = 10.38	**0.004**
	H'	3.22	2.80	F1,19 = 1.34	0.262
	Non-native Cover	0.05	0.09	F1,19 = 1.73	0.204
	Beta diversity	0.48	0.60	F1,39 = 15.10	**<0.001**
	Floristic Quality	3.51	3.95	F1,19 = 2.71	0.116
	Obligate cover	0.13	0.34	F1,19 = 10.30	**0.005**
	OBL+ FACW cover	0.29	0.61	F1,19 = 18.82	**<0.001**
	FACU+UPL cover	0.49	0.21	F1,19 = 11.80	**0.003**
**B) East Marsh**					
Bahiagrass pasture	SR	7.08	7.62	F1,59 = 1.15	0.289
	H'	2.47	3.51	F1,59 = 15.41	**<0.001**
	Non-native Cover	0.47	0.46	F1,59 = 0.08	0.786
	Beta diversity	0.50	0.50	F1,119 = 0.39	0.950
	Floristic Quality	2.52	2.82	F1,59 = 5.25	**0.026**
	OBL cover	0.09	0.18	F1,59 = 9.10	**0.004**
	OBL+ FACW cover	0.20	0.27	F1,59 = 3.63	0.062
	FACU+UPL cover	0.47	0.48	F1,59 = 0.05	0.827
					
Shallow marsh	SR	12.23	11.08	F1,59 = 6.64	**0.013**
	H'	6.04	6.27	F1,59 = 0.35	0.557
	Non-native Cover	0.03	0.02	F1,59 = 0.89	0.349
	Beta diversity	0.57	0.60	F1,119 = 6.28	**0.017**
	Floristic Quality	3.67	4.03	F1,59 = 15.94	**<0.001**
	OBL cover	0.25	0.34	F1,59 = 9.73	**0.003**
	OBL+ FACW cover	0.59	0.63	F1,59 = 0.87	0.354
	FACU+UPL cover	0.02	0.06	F1,59 = 12.56	**0.001**
					
Sawgrass marsh	SR	10.98	10.18	F1,59 = 3.18	0.080
	H'	6.01	5.21	F1,59 = 5.45	**0.023**
	Non-native Cover	0.03	0.002	F1,59 = 8.01	**0.006**
	Beta diversity	0.55	0.47	F1,119 = 19.49	**<0.001**
	Floristic Quality	3.92	4.07	F1,59 = 3.70	0.059
	OBL cover	0.51	0.56	F1,59 = 2.04	0.159
	OBL+ FACW cover	0.67	0.76	F1,59 = 5.75	**0.020**
	FACU+UPL cover	0.02	0.02	F1,59 = 0.18	0.677
					
Wet prairie	SR	17.17	14.83	F1,29 = 9.77	**0.004**
	H'	8.95	7.31	F1,29 = 3.74	0.063
	Non-native Cover	0.03	0.03	F1,29 = 0.22	0.643
	Beta diversity	0.52	0.55	F1,59 = 1.99	0.150
	Floristic Quality	4.04	3.94	F1,29 = 0.45	0.508
	OBL cover	0.16	0.13	F1,29 = 0.50	0.484
	OBL+ FACW cover	0.39	0.36	F1,29 = 0.35	0.559
	FACU+UPL cover	0.07	0.12	F1,29 = 5.26	**0.029**

Floristic quality (measured as the average coefficient of conservatism in the quadrat) increased following restoration in all but the wet prairie community. This increase was significant in the East Marsh bahiagrass pasture and in the shallow marsh communities in both the East Marsh and the South Marsh easements (Table 3). When species cover was used to calculate floristic quality (weighted average coefficient of conservatism), only the bahiagrass community from the East Marsh and the shallow marsh community from the South Marsh showed an increase in floristic quality (S3 Fig).

**Fig 3:**
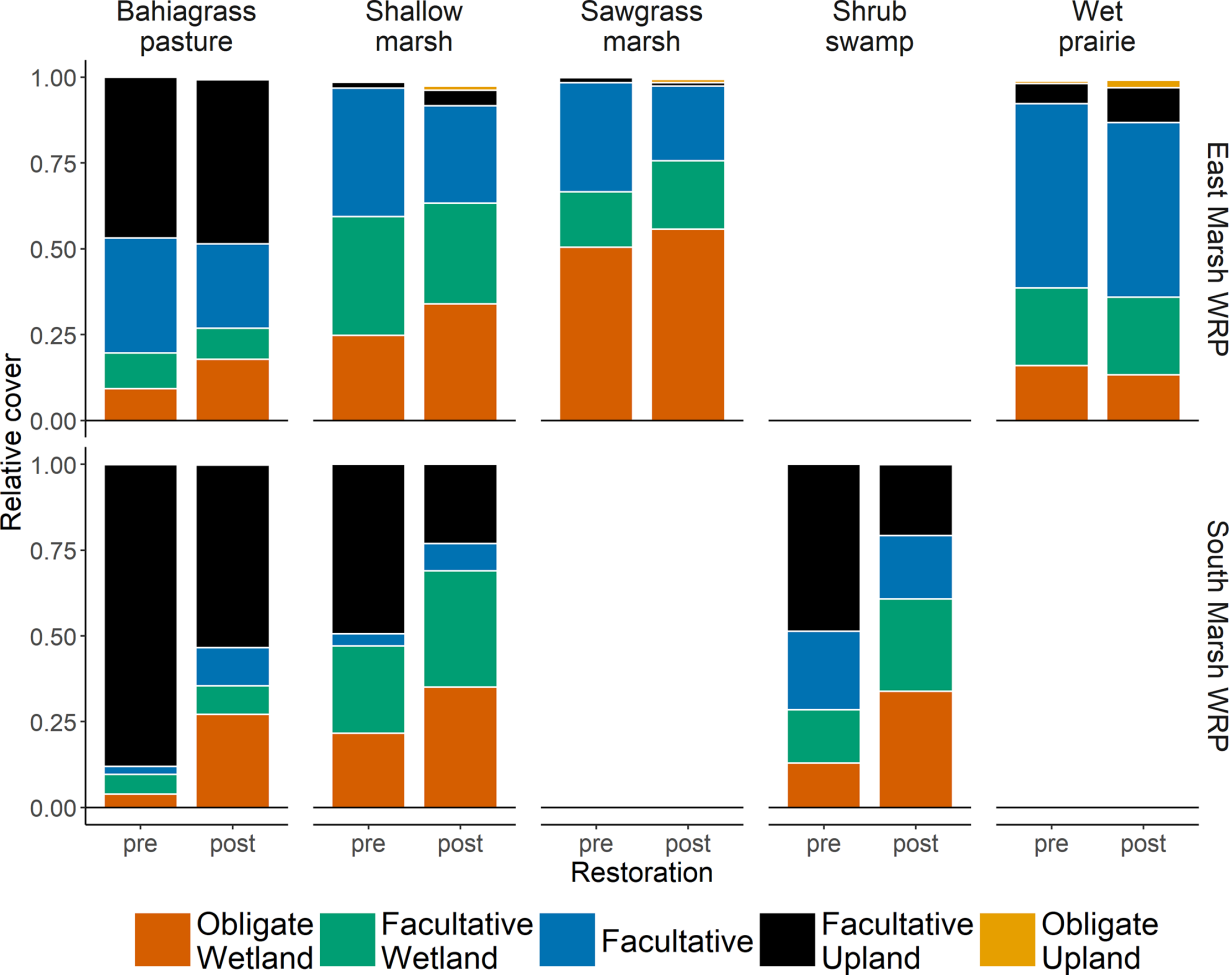
Average relative cover of each wetland species categories in each community type prior and after restoration.

Cover of obligate wetland species (OBL) and facultative upland (FACU) species varied among communities (Fig 3, S4 Fig). Before restoration bahiagrass pastures were characterized by low cover of obligate wetland species (3.9% in the South Marsh, 9.2% in the East Marsh) and high cover of facultative upland (FACU) and obligate upland (UPL) species (88% in the South Marsh, 46.9% in the East Marsh), whereas sawgrass communities had higher cover of obligate wetland species (50.5% in the East Marsh) and almost no facultative upland species (1.5%). The cover of obligate wetland species increased following restoration in all communities but sawgrass and wet prairies (Table 3, Fig 3, and S4 Fig). This increase was associated with a significant decrease in facultative upland and obligate upland species in the South Marsh and with a decrease in facultative (FAC) species in the East Marsh. The size of the effect was higher in the South Marsh compared to the East Marsh easement. For example, we observed a 597% increase in obligate wetland species cover in the South Marsh compared to a 93.48% increase in the East Marsh. When we combined cover of obligate wetland and facultative wetland species, we observed a significant increase in sawgrass communities following restoration (Table 3, S4 Fig).

Overall *Paspalum notatum* cover decreased following restoration (F_1,108_ = 5.675, p = 0.02). Bahiagrass occurred in 93 plots (cover = 53.83%) before restoration and 84 plots (cover = 43.45%) after restoration. *Panicum hemitomon* occurred in 70 plots before restoration and 78 plots after restoration. Maidencane cover significantly increased following restoration with on average 8.81% before restoration and 14.1% after restoration (F_1,95_ = 8.32, p = 0.005). *Cladium jamaicense* cover increased following restoration. Sawgrass occurred in 56 plots (cover = 13.91%) before restoration and 66 plots (cover = 18.17%) after restoration (F_1,71_ = 17.66, p<0.001).

### Effect of cattle grazing on restoration of vegetation

**Fig 4:**
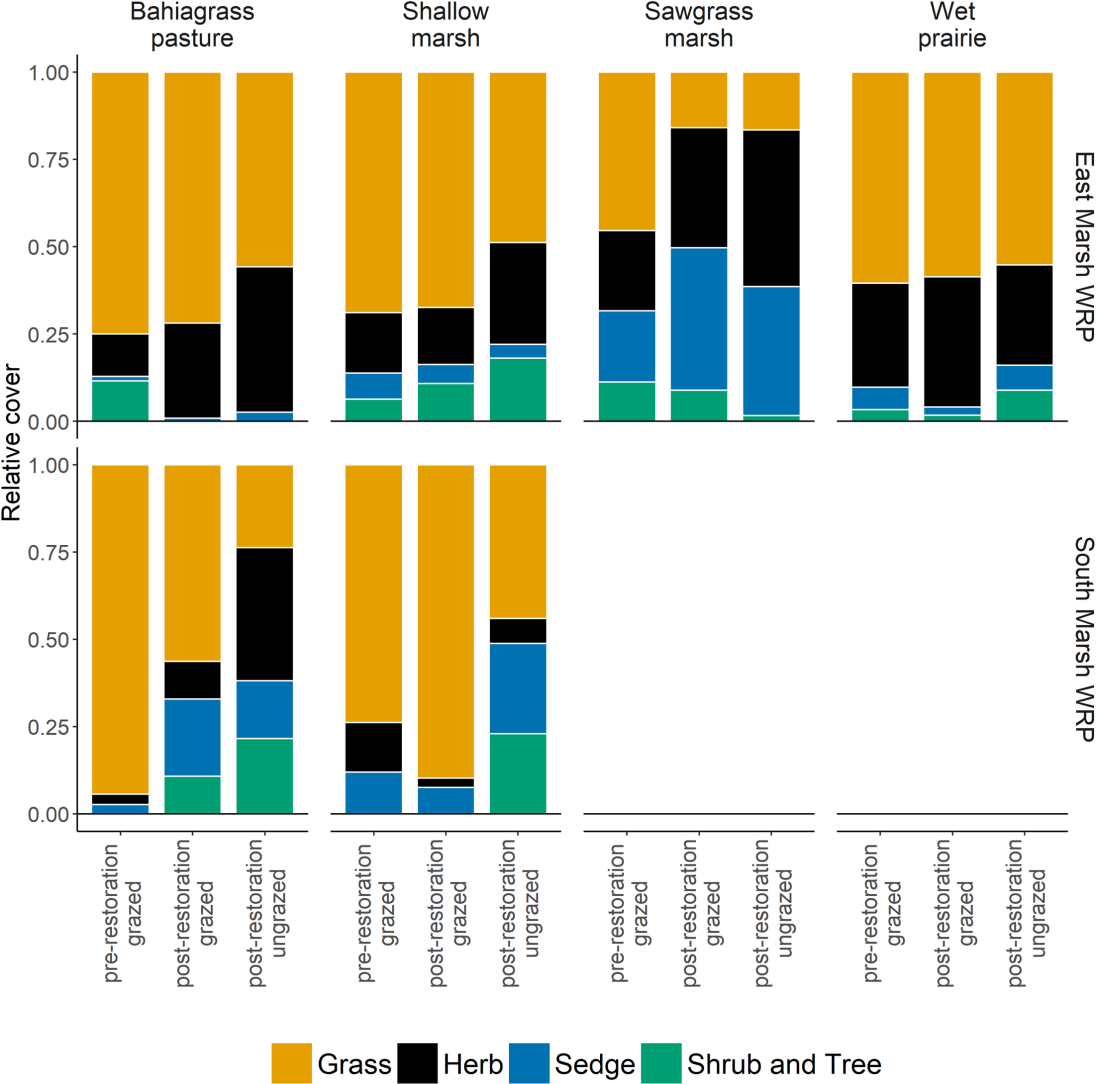
Average relative cover of each life form prior and following restoration and in response to removal of grazing. Before restoration all 300 plots were grazed. We used a subset of the grazed plots for this analysis (see details in material and methods).

The majority of our studied plots (268 out of 300) were grazed by cattle. Nevertheless, as shown in the results above, we still detected increases in floristic quality and obligate wetland species cover in these plots. To further explore grazing effects we compared fenced plots (n_1_ = 38) to nearby grazed plots (n_2_ = 38), and observed a tendency for higher tree and shrub cover in fenced plots, but this increase was not significant (Table 4, Fig 4). We found that grazed plots had higher exotic cover (primarily in bahiagrass community) than nearby fenced plots. Removing cattle significantly increased forb cover, but decreased grass cover especially in bahiagrass pasture and shallow marsh communities (S1 Table). We did not observe a significant effect of removing cattle on species richness and species diversity (H’), despite tendencies for higher diversity in grazed plots.

**Table 4:** Results of linear mixed models testing the effect of removing grazing after restoration. Because sample size was lower, we performed the analysis after combining all community types. No exclosure was setup in the shrub swamp community.

Metrics	Grazed	Fenced	F-value	P-value
SR	9.34	8.76	F1,56 = 0.84	0.363
H	5.06	4.54	F1,56 = 1.33	0.254
Exotic cover	0.16	0.07	F1,56 = 7.49	**0.008**
Grass cover	0.56	0.38	F1,56 = 11.64	**0.001**
Exotic grass cover	0.16	0.06	F1,56 = 7.60	**0.008**
Tree + shrub cover	0.07	0.13	F1,56 = 1.77	0.188
Forb cover	0.21	0.33	F1,56 = 9.77	**0.003**
Sedge cover	0.16	0.16	F1,56 = 0.01	0.946

## Discussion

Monitoring restoration success is a crucial step in ecological restoration. There are multiple reasons why wetland restoration efforts may fail, especially when restoration consists solely in reestablishing hydrology [39]. First, it is possible that the hydrological restoration was unsuccessful at restoring a specific hydrological regime due to poor design, or unexpected groundwater flow [40]. Second, it is possible that plant communities are resistant to traditional restoration efforts and fixed in alternative stable states [41–43]. Third, restoration is a slow process and changes may be observed at later stages of development [39]. Our results suggest this is not the case in our study, as the hydrological restoration partially met the goals of the restoration in both easements. However, the extent of the effect of the restoration depended on community types and easement sites.

### Restoring hydrology

We observed that both median water table and number of flooded days increased following restoration in the South Marsh easement). Following restoration, the average number of flooded days changed from on average (average over 6 wells excluding SM2), to 118.4 days /year in the South Marsh. This is similar to the published natural wet prairie hydroperiod which ranges from 50 to 150 days/year [44], but lower than bay swamp hydroperiod which covered part of the South Marsh easement prior to conversion to pasture. SM2 was the only well inside the South Marsh easement that did not respond positively to the restoration with almost no flooded days in years prior to and post-restoration. Well SM2 behaved similarly to well SM1 which was located just outside of the easement. This can be explained by SM2 being located at a higher elevation (9.30 m above sea level) than any other well within the South Marsh easement, as well as being separated from the rest of the South Marsh by a large ditch isolating this section from the rest of the easement. We detected a tendency for higher water levels and hydroperiods outside of this easement, although this was not significant and essentially due to well SM9. This suggest that the restoration may have had an effect outside of the easement. This is likely due to unforeseen seepage since well SM9 is at low elevation. This is important since a goal of USDA restoration is not to have off-site impacts.

Contrary to the South Marsh, we did not detect any effect of restoration on the hydrology of the East Marsh easement. Number of flooded days were not significantly different prior to and after the restoration, with respectively 169.4 and 146.6 flooded days/year (average from EM1 and EM2 wells). This hydroperiod falls in the high range of typical wet prairies hydroperiod (50–150 days/year). It is possible that the hydrological restoration failed to keep more water within the East Marsh easement. However, we detected a significant increase in the cover of obligate wetland species in the East Marsh following restoration, suggesting this explanation might be incorrect. In addition, water depth and number of flooded days decreased significantly outside the East Marsh easement suggesting that surface flow was retained in the easement which would have flowed into the adjacent pastures prior to restoration. This also suggests that the direct vicinity of the East Marsh easement was impacted by the restoration which may have consequences for land managers. In hindsight, the location of groundwater wells in the East Marsh were likely inappropriate because they were located in lower elevation areas which experienced flooding pre and post restoration. The East Marsh easement has a more heterogeneous topography and fewer ditches compared to the flatter and highly ditched South Marsh. Because of this, the East Marsh always held water at its center despite ditching. In our design, only three groundwater wells (out of nine) were within the boundary of the East Marsh. One well (EM5) was setup in a large ditch and the other two were setup at low elevation (EM1, EM2), where the number of flooded days per year were high even before restoration.

### Restoring plant communities

One of the goals of the hydrological restoration was to increase species alpha and beta diversity. This goal was only partially met. The East Marsh bahiagrass pasture was the only community where we observed an increase in species diversity following restoration. Other communities significantly lost species. However, these patterns were mainly driven by a loss of “rare” species, since most differences became non-significant when species cover was taken into account. This suggests that the effect of hydrological restoration on species alpha diversity was stronger in the most disturbed and species poor vegetation type. Most communities in our study had very low non-native species cover before and after restoration (respectively 3.6% and 3% exotic cover) suggesting that non-native species were not a big problem in our easements. The only exception was bahiagrass pastures community where non-native species cover was high but decreased following restoration in the South Marsh (58% non-native cover before restoration and 32% cover after restoration, mainly related to changes in bahiagrass). We observed a consistent increase in beta diversity in all communities from the South Marsh. This increase in beta diversity suggests that plots within the same community are becoming more dissimilar from each other. Beta diversity has been proposed as a relevant tool to assess restoration success, because it indicates colonization is occurring [45]. In the East Marsh, beta diversity also increased in shallow marsh but remained stable in the wet prairie community and decreased in sawgrass community. The wet prairie community was the most diverse plant community in our study and was relatively pristine before restoration. It is thus likely that this community will not change or that it will take more time to see effects of the restoration. Following restoration, the sawgrass community showed a greater dominance by sawgrass (*Cladium jamaicense*)and in turn led to a more homogenous and species poor community [44]. This demonstrates that restoration may increase or decrease beta diversity depending on the plant community under scrutiny.

Floristic quality (measured as the mean coefficient of conservatism) significantly increased in response to restoration in shallow marshes and in bahiagrass pastures of both easements. Although not significant, we also observed a tendency towards higher floristic quality after restoration in sawgrass marsh and shrub swamp communities. Floristic quality increased primarily because the number and the cover of generalist species (*i*.*e*. species with a coefficient of conservatism of 0, 1, and 2, *e*.*g*. *Centella asiatica*, *Eleocharis vivipara*) decreased following restoration (S3 Fig). Changes in floristic quality were not due to increases in the number and cover of specialist species (i.e. species with coefficient of conservatism >5, *e*.*g*. *Ludwigia suffruticosa*, *Rhynchospora inundata*). This suggests that restoration primarily reduced the cover of non-specialist species (i.e. species with coefficient of conservatism <5) and has yet to have an effect on habitat specialists. Coefficient of conservatism scores used to calculate floristic quality are allocated based on the knowledge of experts and thus come with limitations. Coefficient of conservatism has been extensively field tested in some regions of the USA[46–48] but their application in the southeastern region of the USA is relatively recent [49]. Although imperfect, coefficient of conservatism is a valuable tool to study plant community integrity and effects of restoration.

As expected, we observed an increase in obligate wetland species cover in most communities. This increase was especially strong in plant communities in the South Marsh easement. Our results are in agreement with results obtained by Toth [50] who found rapid increase in obligate wetland species cover in restored sites along the Kissimmee River in Florida. The increase in obligate wetland species observed in the South Marsh coincided with a decrease in facultative upland species, again in agreement with Toth [49]. By contrast, in the East Marsh the increase in wetland species was associated with a decrease in facultative species. Only the sawgrass and wet prairie communities did not significantly gain more obligate wetland species. In the case of the sawgrass community, we observed a significant increase, when both obligate and facultative wetland species were combined. This suggests that the sawgrass community was also positively affected by the restoration despite being already dominated by facultative wetland and obligate wetland species before the restoration.

Another goal of the restoration was to reduce cover of Bahiagrass and increased cover of maidencane and sawgrass. This objective was met. Bahiagrass cover decreased following restoration, whereas maidencane and sawgrass increased following restoration. In fact, we did not detect bahiagrass in several plots previously dominated by this species. Maidencane can sometimes form monospecific stands, especially in the absence of grazing. This is not the case in our study since maidencane cover was only on average 15% after restoration, even when cattle were excluded from plots. This suggests that maidencane has not fully recovered four years after hydrological restoration.

### True effect of restoration or consequences of a shift in rainfall pattern

One limitation of our study is the lack of control plots for vegetation surveys and a dataset including only two survey periods (one before restoration and one after restoration). One may argue that we cannot truly attribute the changes observed to restoration itself. This a fair criticism. Indeed, our results could also be the result of increases in annual rainfall (a major driver of wetland hydroperiod in central Florida) between the two surveys, which would select for species better adapted to wetter conditions. However, our analysis of rainfall pre- and post- restoration showed there was not a significant difference in annual rainfall between the two surveys. Annual rainfall was on average 109.3±27.7cm before the first survey (2000–2005) and 111.2±22.5cm between 2006 and 2012. For these reasons, we argue that the changes in plant vegetation were due to restoration itself and not to shift in annual rainfall.

### Importance of past management

In this study, the strongest responses were observed in the most converted bahiagrass and shallow marsh communities whereas relatively pristine wet prairie did not respond to restoration. Moreover, the success of restoration was especially strong in the South Marsh where conversion to pasture and drainage was more intense than in the East Marsh [30]. The stronger responses to restoration in the more converted South Marsh easement suggests that disturbed sites may benefit from restoration more than less disturbed sites, such as the East Marsh. However, our study focused on restored wetlands within semi-native pastures, which are historically less managed than improved pastures and have never been exposed to fertilizer additions [31]. The big differences between wetlands embedded in improved pastures and semi-native pastures are higher nutrients and higher cover of non-native species [27,51,52], which may impede and/or alter trajectories following restoration [40]. Thus, information is still needed to understand how wetland restoration taking place in improved pastures respond to hydrological restoration.

### Impact of cattle grazing on the success of wetland restoration

Grazing in a wetland restoration easement is a controversial issue [17]. Our results suggest the low-level cattle grazing regime used in this study had a neutral effect on the success of restoration. Indeed, grazing had neither a negative effect nor a clear positive effect on the success of restoration. 262 out of 300 plots were grazed, but we still observed higher obligate wetland species cover, higher floristic quality and higher beta diversity in many plant communities following restoration. By comparing fenced plots to nearby grazed plots, we observed that cattle grazing slowed the decrease of bahiagrass following restoration. We also found that forbs significantly increased and tree and shrub cover slightly increased when grazing was removed, a pattern also observed in fenced seasonal depressional wetlands on Buck Island Ranch [27] and in other herbaceous dominated ecosystems [53–56]. Tree and shrub encroachment is a worldwide phenomenon [57,58] that may reduce landscape heterogeneity, biodiversity, and modify soil properties, ecosystems functions and services [57]. Ranch managers are required to follow strict guidelines when grazing restored wetlands that require grazing at low stocking density, but it is likely that prolonged and higher stocking densities would be detrimental to wetland restoration success. Thus, future research should focus on determining grazing thresholds [59] and testing the effect of seasonality of grazing. Additionally, fire could be used as an ecologically sound alternative to grazing to reduce tree encroachment in restored easement. This could be particularly important for wetland species that are highly preferred by cattle such as *Panicum hemitomon*.

## Conclusions

With 2.3 million acres enrolled, the USDA NRCS Wetland Reserve Easement Program is among the largest restoration programs in the USA. For government officials, the success of the program lies in its capacity to involve private landowners. While acreage enrolled is a sign of a success story, real success is achieved only if wetland habitats and wetland communities are responding positively to restoration. Here, we provide evidence that hydrological restoration improved wetland hydroperiod in one of the two conservation easements. Restored hydroperiods at both sites were within the range of natural wetland communities of the same type. More importantly, we observed that both easements had an increase in floristic quality, beta diversity, and obligate wetland species cover and these benefits occurred with an approved cattle grazing regime. These beneficial effects were particularly strong in the highly modified/managed bahiagrass pastures communities and the ecotone shallow marsh communities which were the most altered by previous agricultural management. The South Marsh easement had the strongest response to the restoration compared to the less disturbed East Marsh. Although these results points to a step in the right direction, the restored communities have not yet fully recovered since particularly important wetland species (*e*.*g*. *Panicum hemitomon*) were present at low abundance. Future work should focus on long-term changes in the vegetation of these easements as well as ecosystem functions.

## Supporting information

S1 Fig **Number of flooded days before and after wetland restoration in the South Marsh (a) and East Marsh (b) easements**. Total cumulative rainfall for each year was plotted as a covariate. Cross indicates years for which we could not reliably assess number of flooded days to due to high numbers of missing data. SM1 and SM9 are located outside of the easements to assess off-site impacts.
(DOCX)

S2 Fig **Species richness (left panel) and species diversity (right panel) response to restoration in each community type and in each restoration easement.** Species diversity was measured using the exponential of Shannon diversity index.
(DOCX)

S3 Fig Floristic quality in response to wetland restoration. **A)** Floristic Quality Index was measured as the mean coefficient of conservatism observed in each plot (left panel) and as weighted mean coefficient of conservatism accounting for specie cover in each plot (right panel).
**B)** Proportion of generalist species (*i*.*e*. species with coefficient of conservatism ≤3 / total number of species in each plot) in response to restoration in each community type and in each restoration easement (left panel). Proportion of specialist species (*i*.*e*. species with coefficient of conservatism ≥7 / total number of species in each plot) in response to restoration in each community type and in each restoration easement (right panel).
(DOCX)

S4 Fig Average relative cover (±se) of obligate wetland species (left panel) obligate and facultative wetland species (middle panel) and facultative upland species (right panel) in each community type, prior and after restoration.
(DOCX)

S1 Table Results of linear mixed models testing the effect of removing grazing in each restored community type. We combined bahiagrass communities from the South Marsh and the East Marsh, and combined Shallow marsh communities from the South Marsh and the East Marsh. Wet prairie is not included because sample size was too small (n = 4). The average of each metric under investigation is reported for grazed and fenced plots.
(DOCX)
