# Supplementary material for: Assessing the success of hydrological restoration in two conservation easements within Central Florida ranchland
Source: PLoS One. 2018 Jul 3;13(7):e0199333. doi: 10.1371/journal.pone.0199333 (PMC6029772; doi:10.1371/journal.pone.0199333)

**S1 Fig:** Numbers of flooded days before and after wetland restoration in the South Marsh (a) and East Marsh (b) easements. Total cumulative rainfall for each year was plotted as a covariate. Cross indicates years for which we could not reliably assess number of flooded days to due to high numbers of missing data. SM1 and SM9 are located outside of the easements to assess off-site impacts.


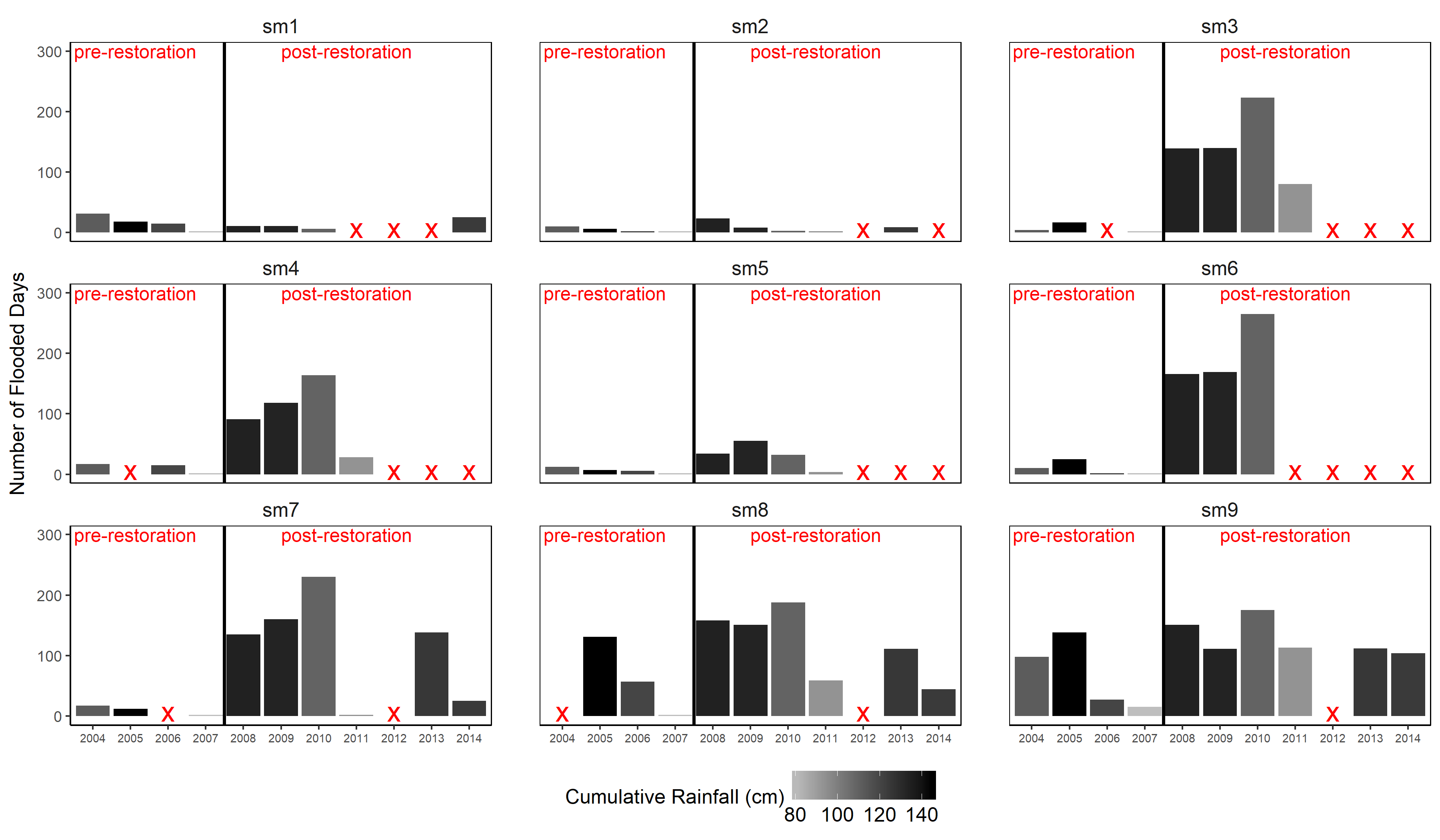

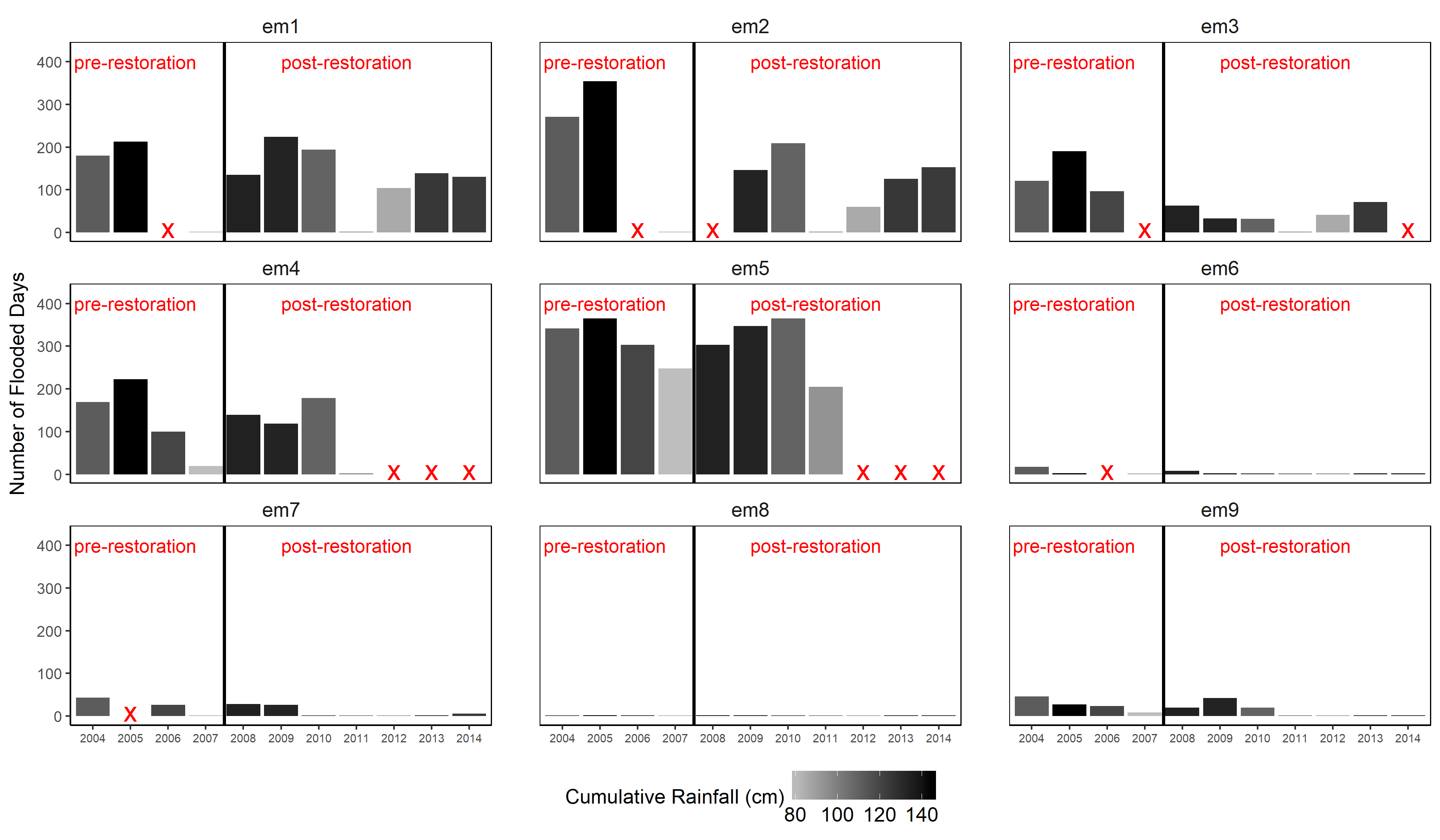

Supplement: S1 Fig — Number of flooded days before and after wetland restoration in the South Marsh (a) and East Marsh (b) easements. Total cumulative rainfall for each year was plotted as a covariate. Cross indicates years for which we could not reliably assess number of flooded days to due to high numbers of missing data. SM1 and SM9 are located outside of the easements to assess off-site impacts. (DOCX) [file pone.0199333.s001.docx]
