# Supplementary material for: Assessing the success of hydrological restoration in two conservation easements within Central Florida ranchland
Source: PLoS One. 2018 Jul 3;13(7):e0199333. doi: 10.1371/journal.pone.0199333 (PMC6029772; doi:10.1371/journal.pone.0199333)

**S2 Fig:** Species richness (left panel) and species diversity (right panel) response to restoration in each community type and in each restoration easement. Species diversity was measured using the exponential of Shannon diversity index.
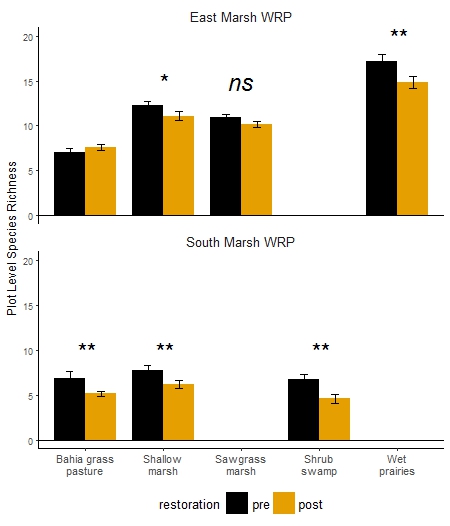

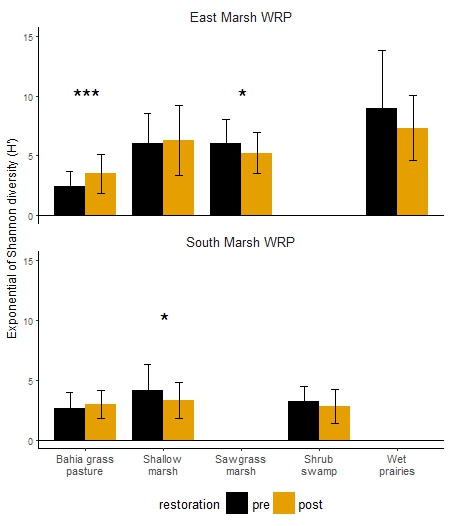

Supplement: S2 Fig — Species richness (left panel) and species diversity (right panel) response to restoration in each community type and in each restoration easement. Species diversity was measured using the exponential of Shannon diversity index. (DOCX) [file pone.0199333.s002.docx]
