# Supplementary material for: Assessing the success of hydrological restoration in two conservation easements within Central Florida ranchland
Source: PLoS One. 2018 Jul 3;13(7):e0199333. doi: 10.1371/journal.pone.0199333 (PMC6029772; doi:10.1371/journal.pone.0199333)

**S3 Fig:** **A)** Average Floristic Quality index (±se) response to restoration in each community type and in each restoration easement. Floristic Quality Index was measured as the mean coefficient of conservatism observed in each plot (left panel) and as weighted mean coefficient of conservatism accounting for species cover in each plot (right panel).


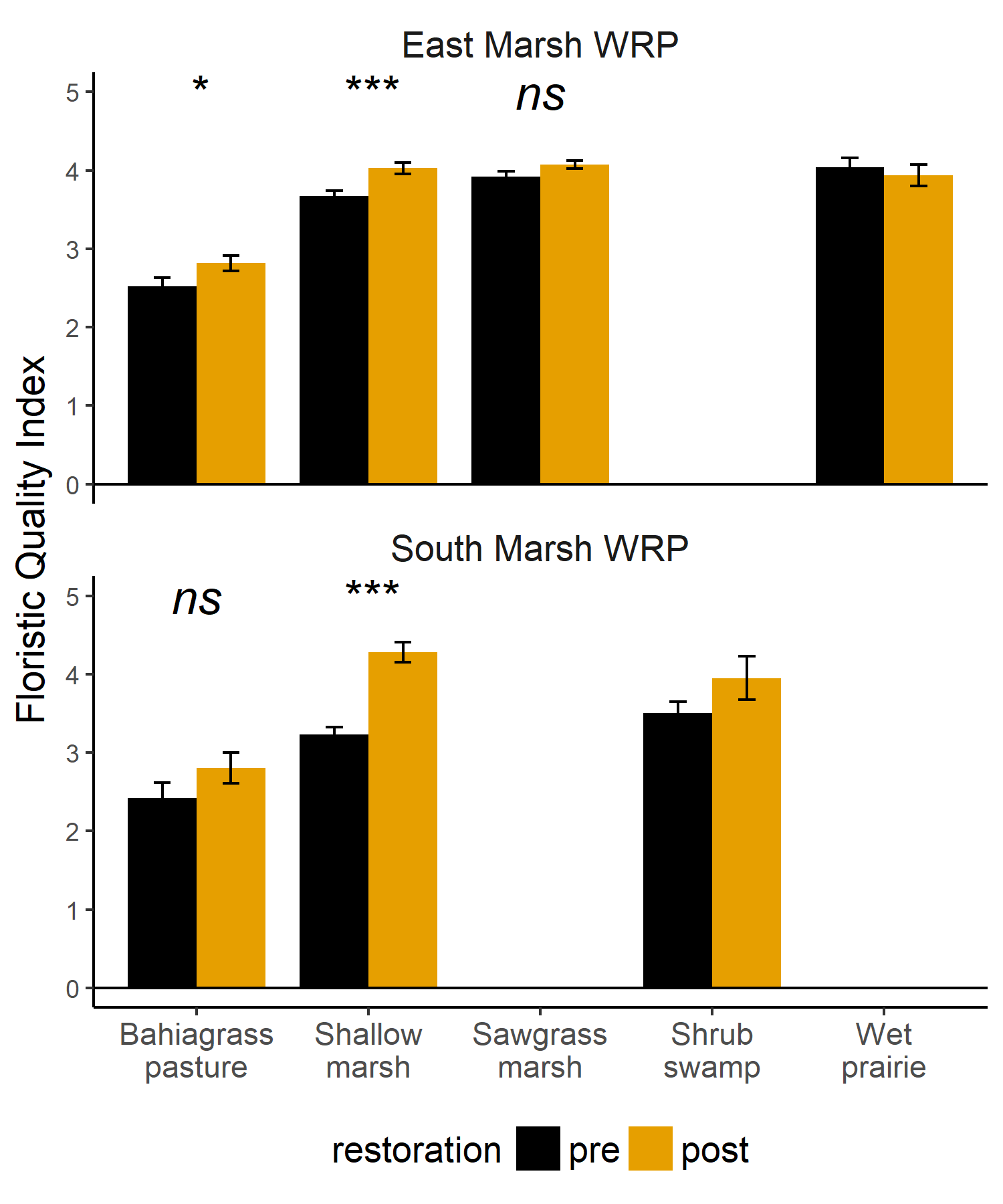


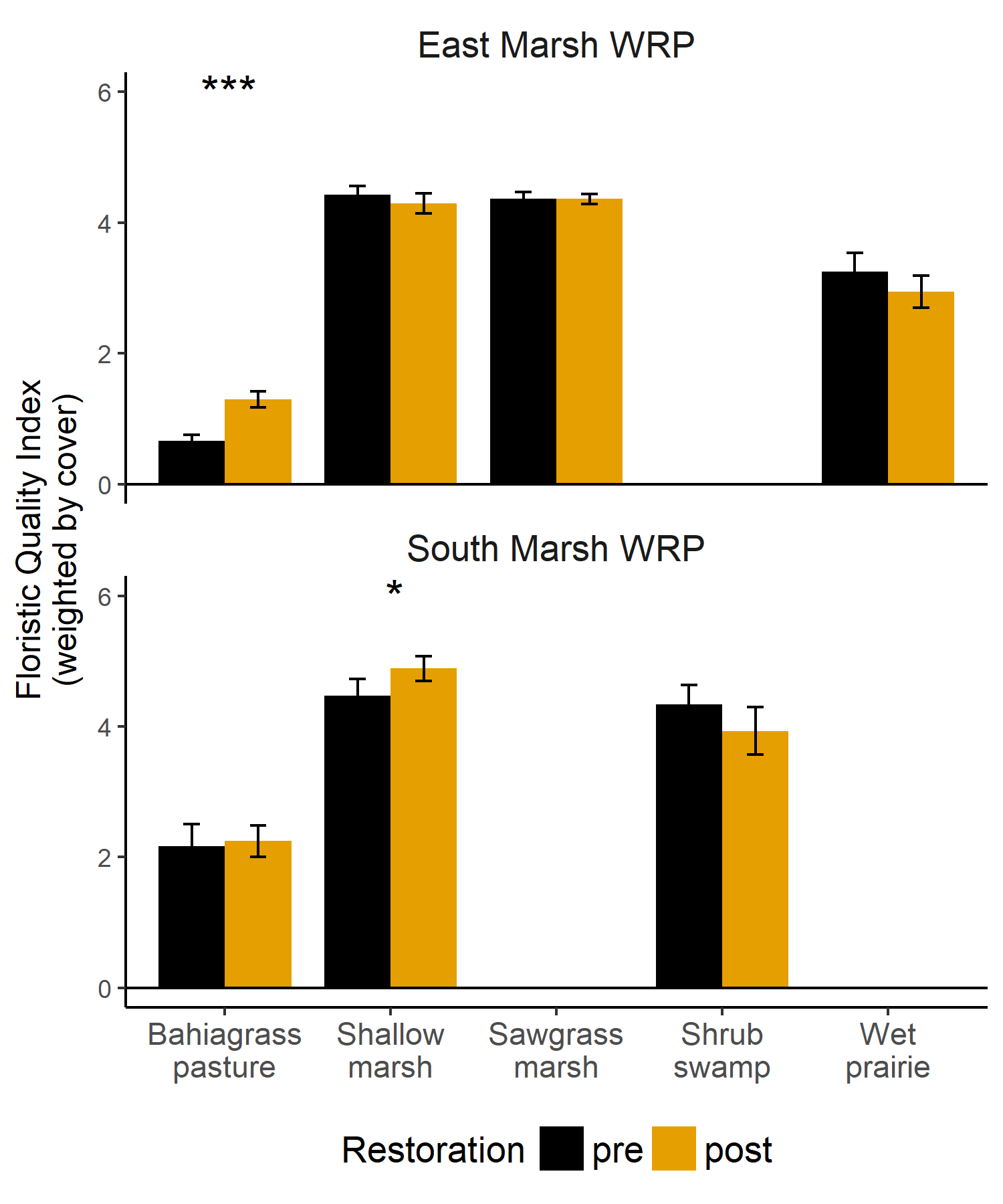


**B)** Proportion of generalist species (*i.e.* species with coefficient of conservatism ≤3 / total number of species in each plot) in response to restoration in each community type and in each restoration easement (left panel). Proportion of specialist species (*i.e.* species with coefficient of conservatism ≥7 / total number of species in each plot) in response to restoration in each community type and in each restoration easement (right panel).


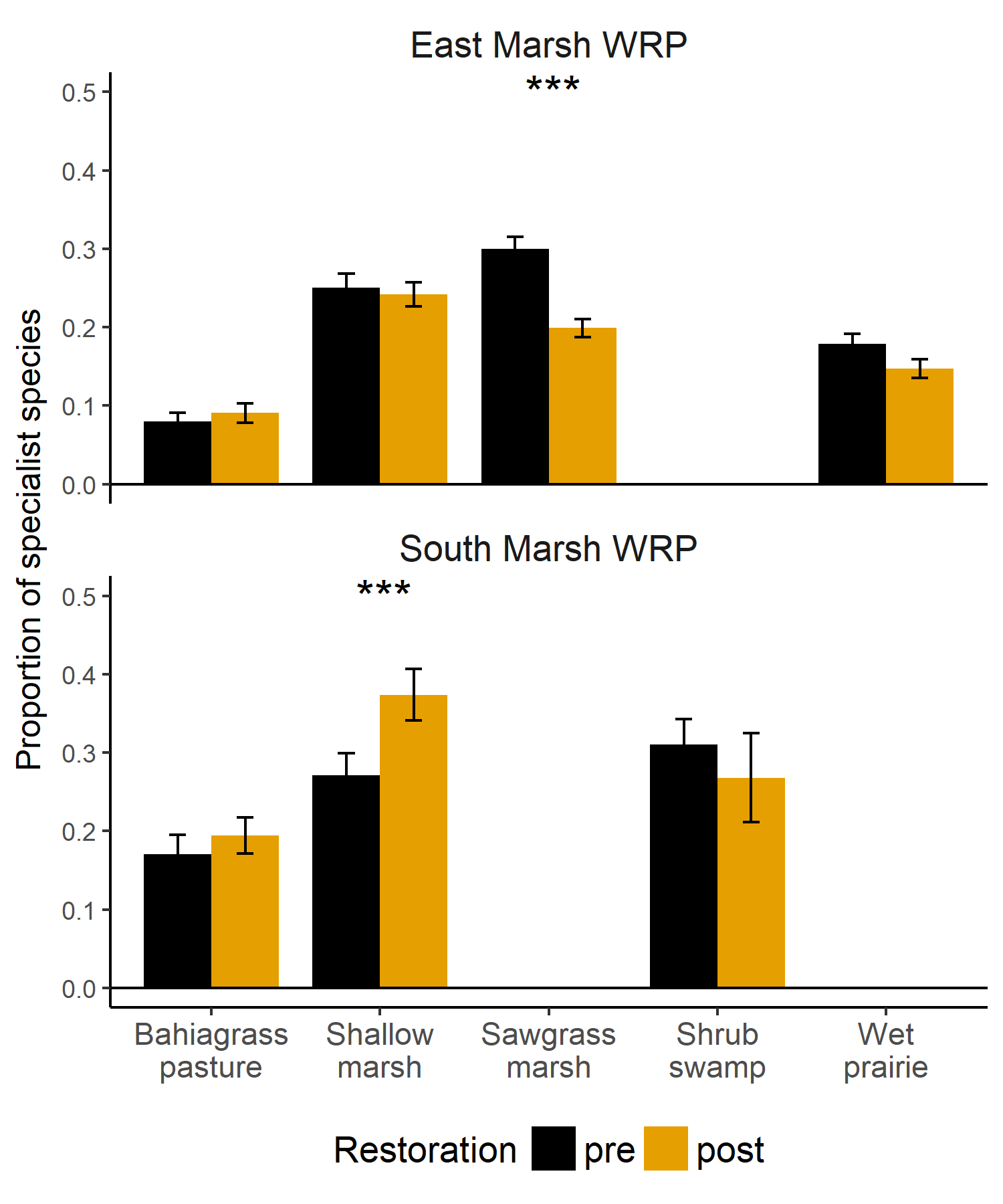

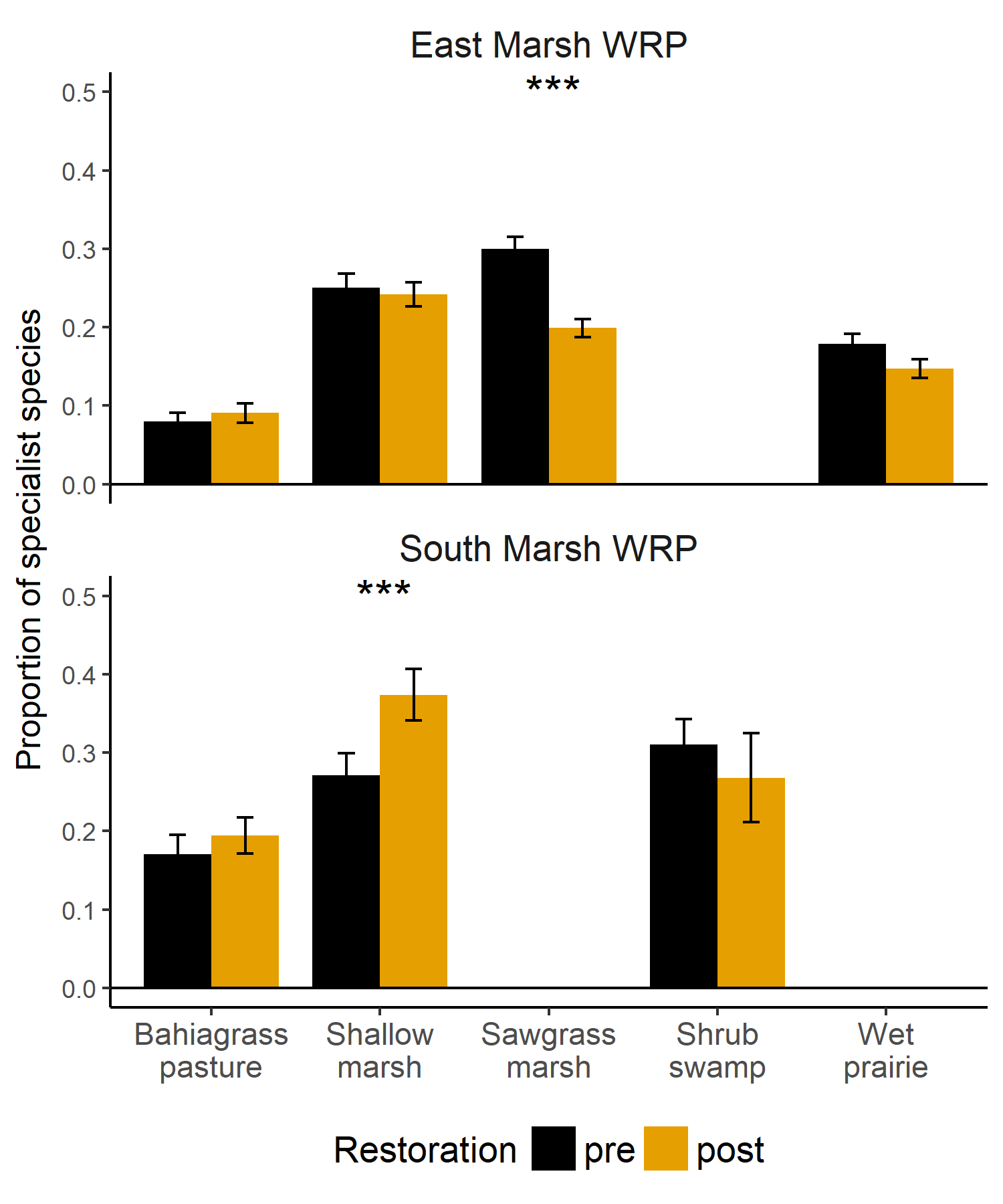

Supplement: S3 Fig — A) Floristic Quality Index was measured as the mean coefficient of conservatism observed in each plot (left panel) and as weighted mean coefficient of conservatism accounting for specie cover in each plot (right panel). B) Proportion of generalist species (i.e. species with coefficient of conservatism ≤3 / total number of species in each plot) in response to restoration in each community type and in each restoration easement (left panel). Proportion of specialist species (i.e. species with coefficient of conservatism ≥7 / total number of species in each plot) in response to restoration in each community type and in each restoration easement (right panel). (DOCX) [file pone.0199333.s003.docx]
