# Supplementary material for: Assessing the success of hydrological restoration in two conservation easements within Central Florida ranchland
Source: PLoS One. 2018 Jul 3;13(7):e0199333. doi: 10.1371/journal.pone.0199333 (PMC6029772; doi:10.1371/journal.pone.0199333)

**S4 Fig.** Average relative cover (±se) of obligate wetland species (left panel) obligate and facultative wetland species (middle panel) and facultative upland species (right panel) in each community type, prior and after restoration.


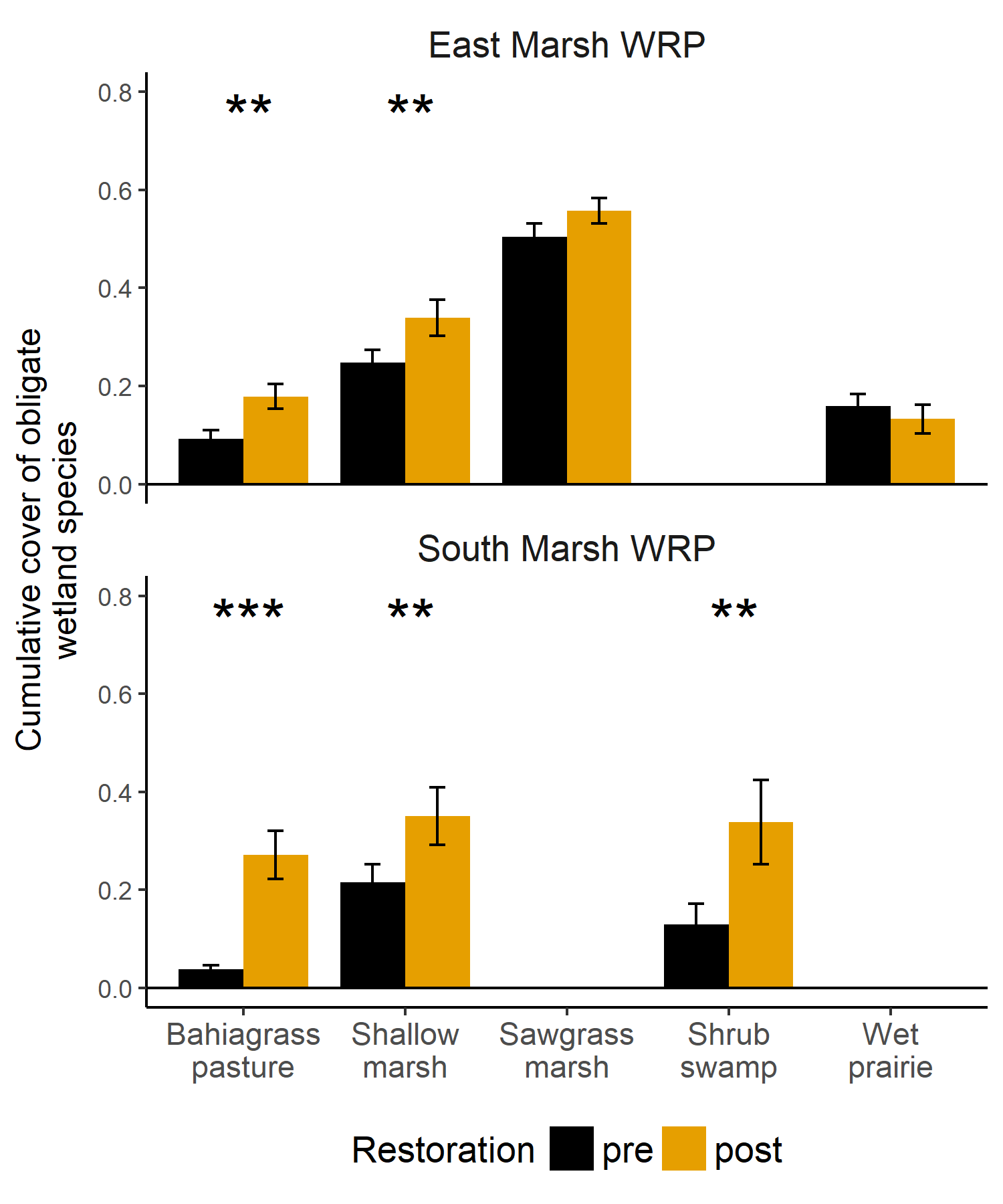

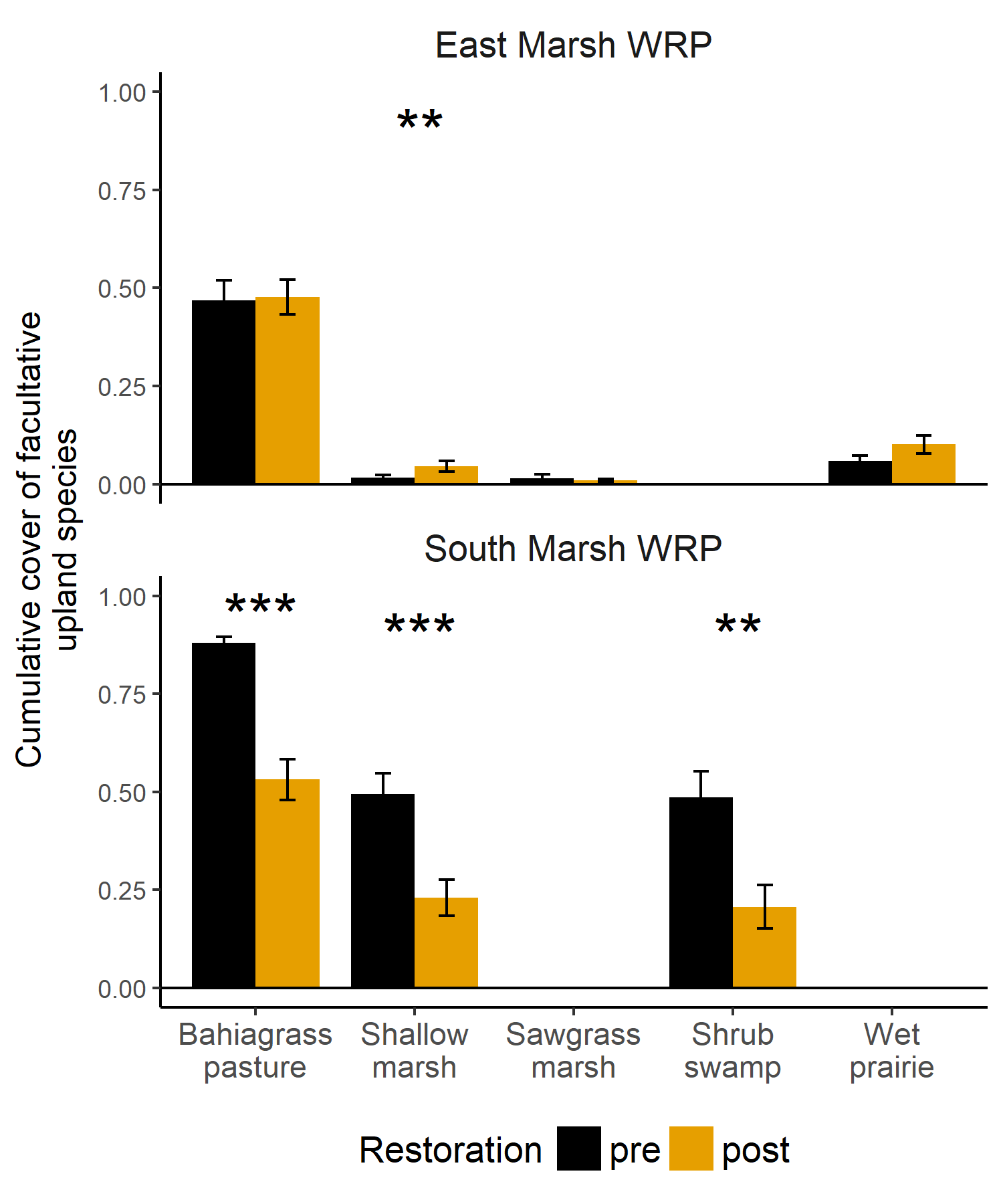


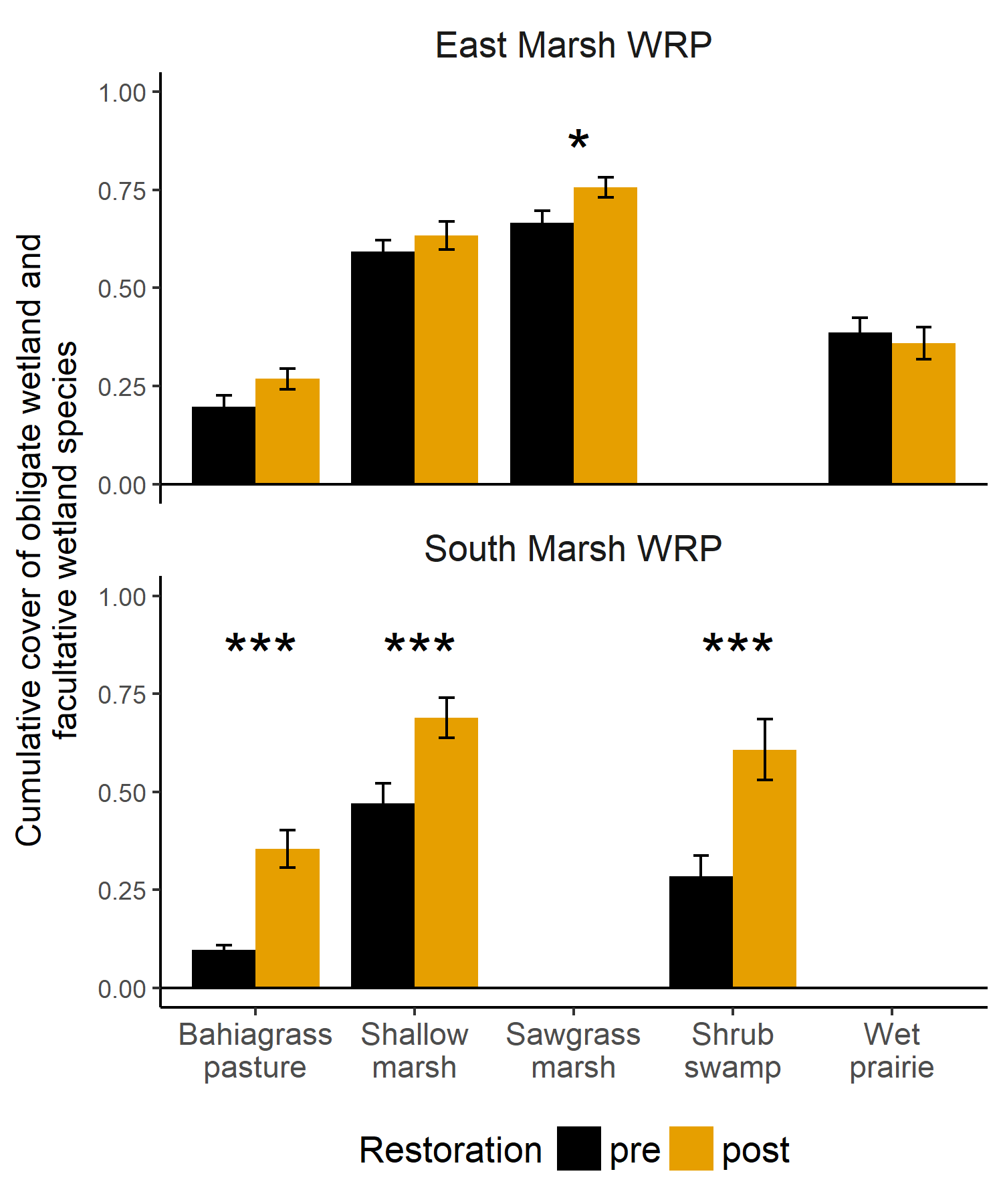

Supplement: S4 Fig — Average relative cover (±se) of obligate wetland species (left panel) obligate and facultative wetland species (middle panel) and facultative upland species (right panel) in each community type, prior and after restoration. (DOCX) [file pone.0199333.s004.docx]
