# Supplementary material for: Assessing the success of hydrological restoration in two conservation easements within Central Florida ranchland
Source: PLoS One. 2018 Jul 3;13(7):e0199333. doi: 10.1371/journal.pone.0199333 (PMC6029772; doi:10.1371/journal.pone.0199333)

**S1 Table.**

A) Results of linear mixed models testing the effect of removing grazing in each community type. We combined Bahiagrass communities from the South Marsh and the East Marsh, and combined Shallow marsh communities from the South Marsh and the East Marsh. Wet prairie is not included because sample size was too small (n=4). The average of each metric under investigation is reported for grazed and fenced plots.

| **Metrics** | **Community types** | **Grazed** | **Fenced** | **F-value** | **P-value** |
| --- | --- | --- | --- | --- | --- |
| SR | Bahiagrass pasture | 7 | 6.667 | F1,17=0.101 | 0.755 |
|  | Shallow marsh | 8.929 | 8.286 | F1,20=0.404 | 0.532 |
|  | Sawgrass marsh | 10.5 | 9.25 | F1,11=0.758 | 0.403 |
| H | Bahiagrass pasture | 3.483 | 3.301 | F1,17=0.074 | 0.789 |
|  | Shallow marsh | 5.28 | 4.285 | F1,20=1.136 | 0.299 |
|  | Sawgrass marsh | 5.64 | 4.409 | F1,11=2.872 | 0.118 |
| Exotic cover | Bahiagrass pasture | 0.448 | 0.202 | F1,17=8.585 | **0.009** |
|  | Shallow marsh | 0.052 | 0.012 | F1,20=0.837 | 0.371 |
|  | Sawgrass marsh | 0 | 0 | NA | NA |
| Grass cover | Bahiagrass pasture | 0.615 | 0.344 | F1,17=9.803 | **0.006** |
|  | Shallow marsh | 0.738 | 0.474 | F1,20=5.714 | **0.027** |
|  | Sawgrass marsh | 0.160 | 0.166 | F1,11=0.012 | 0.916 |
| Exotic grass cover | Bahiagrass pasture | 0.436 | 0.200 | F1,17=7.503 | **0.014** |
|  | Shallow marsh | 0.052 | 0 | F1,20=1.341 | 0.261 |
|  | Sawgrass marsh | 0 | 0 | NA | NA |
| Tree + shrub cover | Bahiagrass pasture | 0.072 | 0.144 | F1,17=2.347 | 0.144 |
|  | Shallow marsh | 0.077 | 0.194 | F1,20=1.216 | 0.283 |
|  | Sawgrass marsh | 0.089 | 0.017 | F1,11=2.732 | 0.127 |
| Forb cover | Bahiagrass pasture | 0.162 | 0.393 | F1,17=8.822 | **0.009** |
|  | Shallow marsh | 0.125 | 0.229 | F1,20=2.870 | 0.106 |
|  | Sawgrass marsh | 0.343 | 0.449 | F1,11=2.811 | 0.122 |

**B)** Average (± standard error) relative grass cover in each community type. Before restoration all plots were grazed but are shown in different colors to illustrate initial differences.


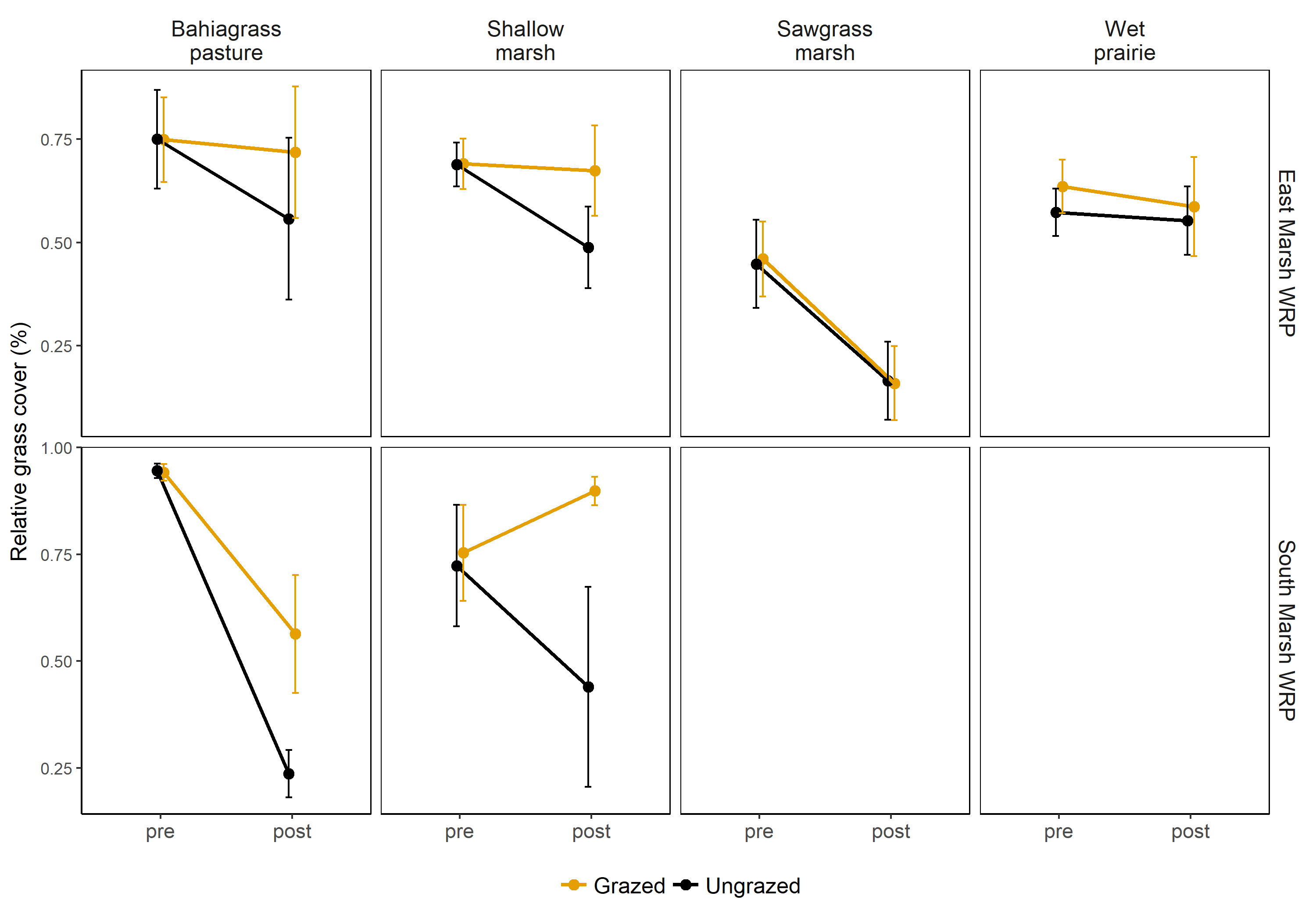


**C)** Average (± standard error) relative tree and shrub cover in each community type. Before restoration all plots were grazed but are shown in different colors to illustrate initial differences.


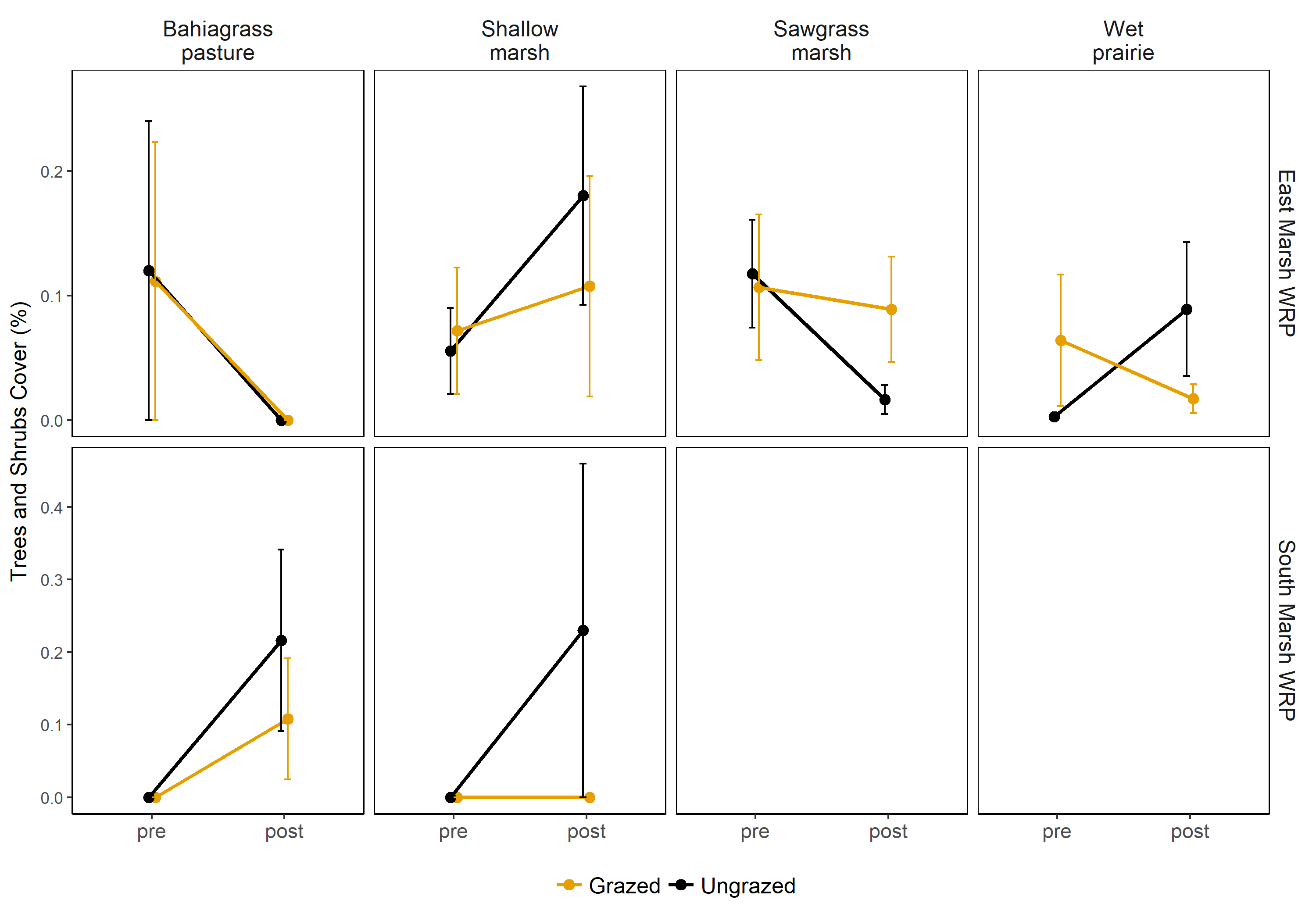


**D**) Average (± standard error) relative exotic grass cover in each community type. Before restoration all plots were grazed but are shown in different colors to illustrate initial differences.


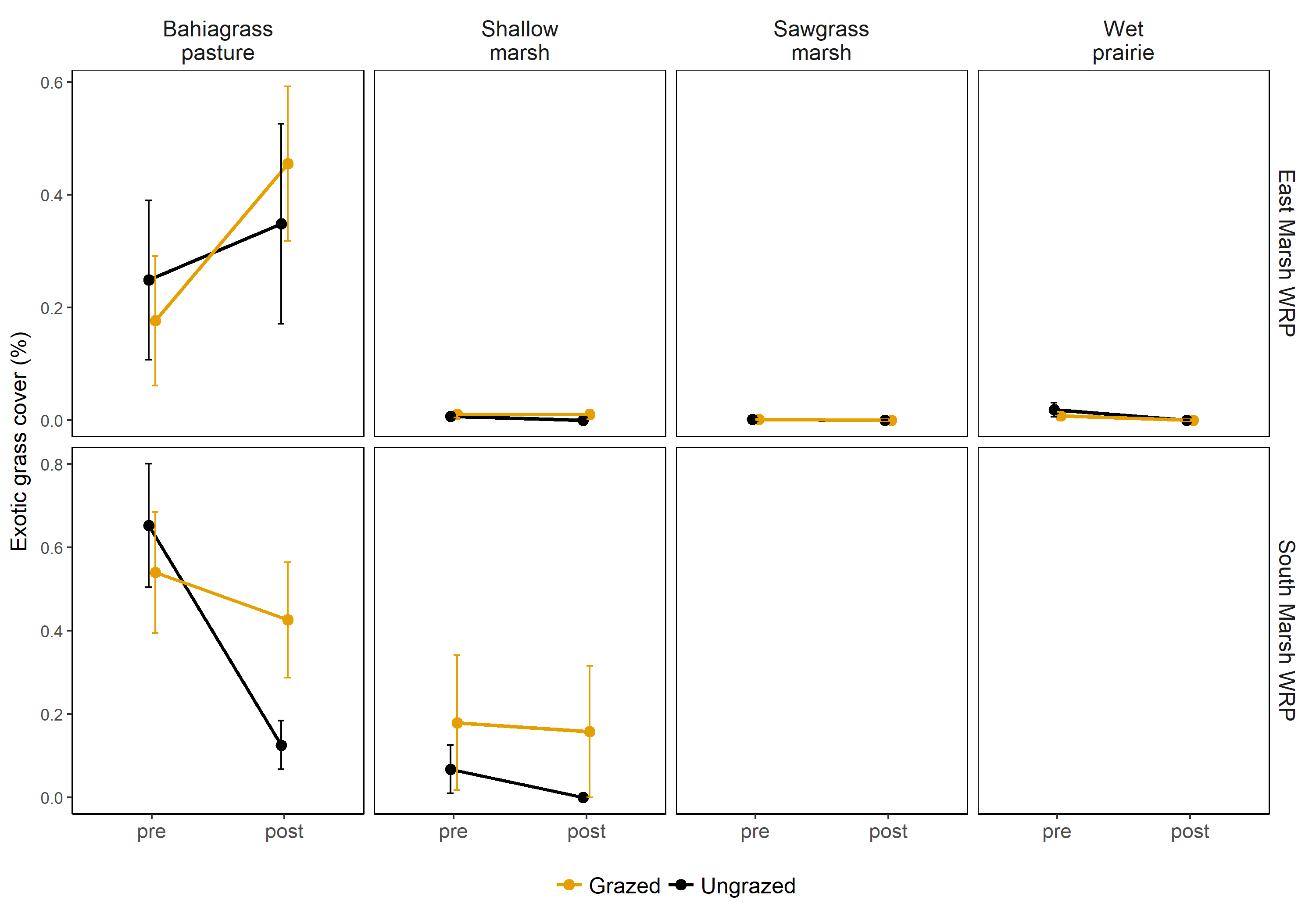

Supplement: S1 Table — We combined bahiagrass communities from the South Marsh and the East Marsh, and combined Shallow marsh communities from the South Marsh and the East Marsh. Wet prairie is not included because sample size was too small (n = 4). The average of each metric under investigation is reported for grazed and fenced plots. (DOCX) [file pone.0199333.s005.docx]
